# Warming Leads to Biomass Increase, Leaf Nitrogen Decline, and Community Turnover in Mediterranean *Nardus stricta* Grasslands

**DOI:** 10.1002/ece3.73537

**Published:** 2026-04-22

**Authors:** Marta Correia, Alexandre Silva, Susana Rodríguez‐Echeverría

**Affiliations:** ^1^ Centre for Functional Ecology (CFE), TERRA Associate Laboratory, Department of Life Sciences University of Coimbra Coimbra Portugal; ^2^ Mediterranean Institute for Advanced Studies (IMEDEA, UIB‐CSIC) Esporles Balearic Islands Spain; ^3^ CISE ‐ Centro de Interpretação da Serra da Estrela, Município de Seia Seia Portugal

**Keywords:** biodiversity, *Festuca* endemic species, high‐elevation pasturelands, Mediterranean climate, mountains, open top‐chamber (OTC), Serra da Estrela, warming

## Abstract

*Nardus stricta* L. grasslands are designated a priority habitat under the European Union Habitats Directive, reflecting their role in biodiversity conservation and soil and water regulation. However, these grasslands are listed as vulnerable in the European Red List of Habitats, primarily due to threats associated with global change. Despite their conservation importance, subalpine *Nardus* grasslands in the Iberian mountains remain poorly studied, and responses to climate warming are largely unknown. We experimentally tested the effects of warming in five semi‐natural *N. stricta* grasslands (1545–1875 m a.s.l.) in Serra da Estrela, Portugal, located at the transition between Mediterranean and Atlantic climates. In 2020, five paired open‐top chambers (OTCs) and control plots were established at each site. Community composition and aboveground biomass were monitored annually from 2021 to 2023, and leaf nutrient content of *N. stricta* was analyzed in 2022. Warming increased mean air temperature by ~1.9°C across sites, reduced freezing exposure, and enhanced thermal accumulation during the growing season. These changes were associated with higher aboveground biomass and increased species richness, accompanied by moderate turnover. The dominant grass *N. stricta* increased in cover across site × year combinations, whereas the snow‐associated endemic *Festuca henriquesii*, restricted to the high‐elevation site COB, declined under warming. Warming also reduced leaf nitrogen concentration in *N. stricta* , altering the nutrient balance of the dominant grass and potentially leading to cascading ecological effects. Responses were site‐dependent, with weaker biomass increases at drier, lower‐elevation sites, suggesting baseline water availability modulated warming effects. Our results demonstrate a consistent thermal signal but context‐dependent ecological responses. Although warming enhanced productivity and richness, it also altered species composition and reduced leaf nitrogen content, suggesting early restructuring of Iberian subalpine grasslands under climate change. These findings highlight the importance of incorporating site‐specific environmental context, particularly soil moisture, into conservation strategies for Mediterranean mountain ecosystems.

## Graphical abstract

Experimental warming in Mediterranean subalpine *Nardus stricta* grasslands increased plant biomass and species richness. However, it drove community turnover, reduced the abundance of cold‐adapted endemics, and lowered leaf nitrogen content, indicating declining forage quality. These responses varied across sites along an elevational and soil‐moisture gradient, largely mediated by local baseline soil moisture, highlighting the vulnerability of this priority habitat under climate change.

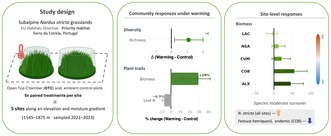

## 1 Introduction

Semi‐natural grasslands are among Europe's most important ecosystems, celebrated for their exceptional biodiversity and the vital ecosystem services they provide (Dengler et al. 2020; Winberg et al. 2024). These services include carbon sequestration, water regulation, soil stabilization, and cultural and social values, alongside their role as critical habitats for diverse flora and fauna (Bengtsson et al. 2019; Richter et al. 2021). Within mountain grasslands, habitats dominated by *Nardus stricta* L., a tussock‐forming perennial grass, hold ecological significance as they support unique plant communities shaped by the harsh climatic conditions of high elevations. Widespread across Europe, *Nardus stricta* grasslands typically develop on acidic, relatively nutrient‐poor soils and have historically been associated with extensive grazing and transhumance practices on common pastures (Leuschner and Ellenberg 2017). These grasslands function as vital reservoirs of biodiversity and actively contribute to soil nutrient dynamics, water cycling, and the provision of food resources. Due to their relevance, *Nardus stricta* grasslands are designated as a priority habitat for conservation under the European Union Habitats Directive, covering 20% of Nature 2000 areas (European Commission 1992, Annex I, Council Directive 92/43/EEC, Habitat Code 6230). However, *N. stricta* mountain grasslands are also threatened due to land‐use changes, grazing abandonment, eutrophication, tourism pressure, and climate change (Galvànek and Janàk 2008; European Environment Agency 2020), and thus are listed as “vulnerable” in the European Red List of Habitats (Janssen et al. 2016).

The average global mean near‐surface temperature for the decade 2015–2024 was about 1.24°C higher than the 1850–1900 baseline, which is the accepted “pre‐industrial” reference period (World Meteorological Organization 2024). This accelerated warming raises significant concerns about the ability of key species to maintain ecosystem balance under changing climatic conditions (Kerr et al. 2015; Parmesan 2006). Mountain ecosystems, including *N. stricta* grasslands, are particularly vulnerable to climate change due to their location near the physiological limits of many species (Körner 2003). This makes them highly sensitive to alterations in temperature and precipitation patterns (Gottfried et al. 2012; Guisan et al. 2019; Sala et al. 2000). In mountain ecosystems, warming may favor thermophilic species at the expense of cold‐adapted specialists, potentially leading to shifts in species composition and declines in biodiversity (Nogués‐Bravo et al. 2008; Pauli et al. 2012). Evidence of global warming's impact is already apparent, with shifts in species distributions, changes in phenological patterns, and disruptions to ecosystem functioning observed in mountain regions worldwide (i.e., Grabherr et al. 1994; Pauli et al. 2012; Peñuelas and Boada 2003; Verrall and Pickering 2020).

Rising temperatures can directly affect plant physiology, phenology, and productivity (Menzel et al. 2006). Warming is expected to reduce the number of days with snow cover, freezing days and extreme frost events, potentially alleviating stress for some species, and promoting growth in others (Wipf et al. 2009). At the same time, warming can influence soil processes, such as nutrient cycling, by altering microbial activity and decomposition rates (Conant et al. 2011). These changes can have cascading effects on plant nutrient uptake, leaf nutrient content, and forage quality (Sterner and Elser 2002). While warming may extend the growing season and promote plant productivity in temperate mountain systems (Perfors et al. 2003; Wang et al. 2020), these positive effects may be counterbalanced in Mediterranean mountains by an exacerbated water stress (Nogués‐Bravo et al. 2008; Escudero et al. 2012; García‐Fernández et al. 2012; Pugnaire et al. 2020). Indeed, complex responses of plant species and plant communities to warming have been observed in Mediterranean mountains (García‐Cervigón Morales et al. 2012; Pauli et al. 2012; Rudley et al. 2023), underscoring the need for site‐specific research on the impacts of climate change in these ecosystems.

Serra da Estrela, the westernmost end of the Iberian Central System and the highest mountain range in mainland Portugal, functions as a transitional zone between the Temperate and Mediterranean climates, with the upper part being characterized as an orotemperate submediterranean climate variant (Jansen 2002; Rivas‐Martínez et al. 2011). Mountains in the transition of these climates are predicted to suffer the greatest loss in plant diversity due to climate change (Thuiller et al. 2005). Thus, this unique biogeographic positioning offers an ideal setting to investigate the effects of climate change on high‐altitude ecosystems. In fact, warming is evident in this region since the mean annual air temperature in Serra da Estrela has increased from 9°C in 1970 to 10.9°C in 2024, while there is a significant shortening and delay of the snow season since the second half of the 19th century (Mora and Vieira 2025). In Serra da Estrela, *N. stricta* grasslands occur mainly at elevations between 1500 and 1900 m.a.s.l., but the occupied area has declined significantly over the past 30 years, largely due to the cessation of sheep grazing and transhumance activity (Monteiro et al. 2020; Mendes and Mora 2025). These ecosystems are also increasingly threatened by rising temperatures, shifting precipitation patterns and anthropogenic pressure (Carvalho and Marques 2020; Monteiro et al. 2020). Despite these challenges, some shepherds continue traditional summer grazing in *N. stricta* grasslands, supporting the production of the region's distinctive “Serra da Estrela Cheese” while helping to preserve its cultural and ecological heritage (Inácio et al. 2020).

Despite their ecological importance, research on *N. stricta* grasslands in the Mediterranean region remains incomplete. Most studies on climate change impacts in (sub)‐alpine and montane grasslands have focused on temperate regions, where water availability is less limiting (i.e., Gottfried et al. 2012; Rumpf et al. 2025; Verrall and Pickering 2020). In temperate mountains, an expansion of *N. stricta* due to increased competitiveness under a warmer, drier climate is forecasted (Casale and Bocchiola 2022; Movedi et al. 2023). Also, shifts in community composition and diversity have been reported in subalpine grasslands because of acute differences in the response of dominant and rare species to warming and drought (Sebastià et al. 2008). In the Mediterranean Basin, rising temperatures are expected to increase the frequency, severity, and duration of drought events, impacting plant growth and diversity and the distribution of plant communities (Guiot and Cramer 2016; Nogués‐Bravo et al. 2008). Therefore, ecosystems in sub‐Mediterranean and Mediterranean mountains with distinct biogeographic conditions, such as Serra da Estrela, may face unique challenges. Understanding how plant communities respond to climatic constraints in these mountains is crucial for conserving these ecosystems.

In this research, open top‐chambers (OTCs) were used to simulate increased temperatures and examine the effects of warming on *N. stricta* grasslands in Serra da Estrela. OTCs are a widely used tool in climate change research, effectively raising air and soil temperatures while allowing natural environmental conditions to persist (Hollister et al. 2023). Over 3 years, we monitored five grassland sites differing in elevation and local environmental conditions to assess changes in species diversity, plant growth, community turnover, and species‐specific responses. We hypothesized that: (a) warming would have mixed effects on diversity indices, potentially increasing species richness but reducing evenness due to the competitive dominance of certain species; (b) warming would enhance plant growth by extending the growing season and alleviating frost constraints; and (c) warming would reduce leaf N and P concentrations through accelerated nutrient cycling and dilution effects linked to higher growth rates.

## 2 Materials and Methods

### 2.1 Study Sites

Serra da Estrela is the westernmost sector of the Iberian Central System and the highest mountain range in mainland Portugal. It lies at the transition between Atlantic and Mediterranean bioclimates (Meireles 2010; Meireles et al. 2012) and is dominated by siliceous substrates, mainly granite and schist. Its geomorphology reflects the two most recent major glaciations and is characterized by two main plateaus separated by the SSW–NNE‐oriented Alforfa and Zêzere valleys. The Upper Plateau (1993–1450 m), located on the western side of the mountain, forms the highest portion of the massif. It lacks prominent peaks but is bounded by steep scarps with more than 1000 m of relief (Vieira et al. 2021). Across the study area, soils are classified as Umbrisols, characterized by coarse texture, low pH, and high organic matter content (Cardoso 1974; Ramos et al. 2017; IUSS WRB Working Group 2006). Umbrisols typically develop in cool, wet environments where acidity, low temperatures, and surface wetness slow organic matter decomposition (IUSS WRB Working Group 2006).

The combination of climatic and topographic heterogeneity supports a mosaic of montane and subalpine habitats, including grasslands, shrublands, and rocky outcrops, which host a rich diversity of endemic and rare species (Meireles 2010). Serra da Estrela is considered a center of endemism and narrowly distributed taxa (Médail and Quezel 1997; Pinto da Silva and Teles 1980), and represents the southwestern limit for many plant species (Jansen 2002). Owing to its ecological and biogeographic relevance, the area is protected as a Natural Park and as a Site of Community interest under the *Natura 2000* Network. The Upper Plateau is a Biogenetic Reserve designated by the European Council and a Wetland of International Importance protected by the Ramsar Convention.

**FIGURE 1:**
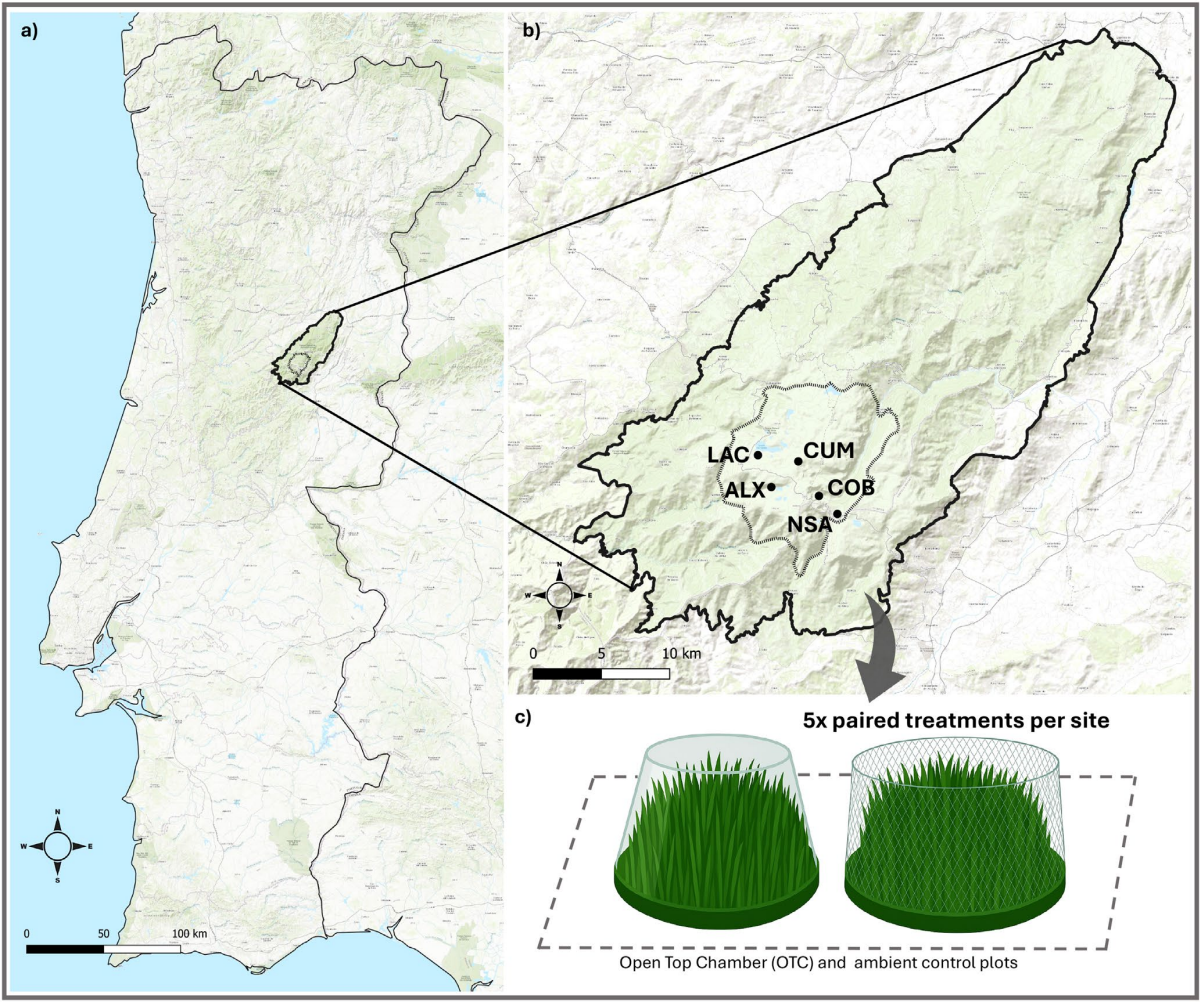
Location of the study area and experimental design. (a) Serra da Estrela Natural Park (PNSE) in mainland Portugal. (b) The Upper Plateau showing the five study sites: Malhõesitos da Talada (ALX), Covão do Boi (COB), Cume (CUM), Lagoa Comprida (LAC), and Nave Santo António (NSA). The solid line indicates the PNSE boundary and the dashed line the Biogenetic Reserve. (c) At each site, five paired plots were established, each consisting of one open‐top chamber (OTC; warming) and one paired fenced control plot (grazing exclusion).

We selected five semi‐natural grasslands (ALX, COB, CUM, LAC, NSA) with *Nardus stricta* as the dominant species spanning the current distribution of these communities in Serra da Estrela (Figure 1, Table 1). ALX and COB have wet, organic, acidic sandy‐loam soils, whereas CUM has a moderately moist loam soil and is comparatively more exposed, with stronger seasonality. NSA is a waterlogged depression with high organic content, while LAC is the warmest and driest site, with the lowest SOM. The study sites reflected two partially independent environmental gradients. First, an elevational–thermal gradient partitioned the three high‐altitude plateau sites (> 1800 m; ALX, COB, CUM; MAT≈7.5°C) from the two lower, warmer sites (NSA and LAC; 8.2 C and 9.3 C, respectively). Second, an edaphic gradient characterized by soil water content (SWC) and soil organic matter (SOM) distinguished the sites into three groups: high (ALX, NSA), intermediate (COB, CUM), and low (LAC) moisture and organic content (Table 1).

**TABLE 1:** Site characteristics, including latitude, longitude, elevation, climate variables (mean annual temperature, MAT; seasonal temperature range, STR), and soil properties (pH, gravimetric soil water content, SWC %; soil organic matter, SOM %; nitrogen content, N % and soil texture). Climate variables were calculated from temperature measurements recorded in ambient control plots over 2 years (2021–2022). Soil properties were measured from samples collected in November 2020 (analytical details in Supporting Information, Appendix S1).

Study sites	Latitude	Longitude	Elevation	MAT	STR	pH	SWC (%)	SOM (%)	*N* (%)	Soil texture
ALX (Malhõesitos da Talada)	40.34114	−7.63745	1823	7.69 ± 1.09	16.00	4.9 ± 0.09	65.3 ± 6.52	44.9 ± 12.37	1.6 ± 0.29	Sandy Loam
COB (Covão do Boi)	40.32393	−7.60237	1870	7.52 ± 1.21	17.91	4.5 ± 0.07	53.5 ± 5.66	35.3 ± 5.21	1.1 ± 0.11	Sandy Loam
CUM (Cume)	40.34533	−7.62245	1875	7.50 ± 1.34	20.85	4.3 ± 0.06	46.9 ± 1.80	35.3 ± 7.49	1.3 ± 0.07	Loam
LAC (Lagoa Comprida)	40.35945	−7.65314	1617	9.29 ± 1.20	17.20	4.9 ± 0.06	40.7 ± 4.89	23.3 ± 3.70	1.1 ± 0.15	Sandy Loam
NSA (Nave Santo António)	40.31482	−7.58218	1546	8.22 ± 1.09	16.99	4.4 ± 0.05	63.7 ± 3.30	44.4 ± 4.37	1.6 ± 0.08	Sandy Loam

All sites are subject to traditional extensive summer grazing, primarily by sheep, and no mowing, fertilization, or other management practices are applied. Experimental plots were protected from herbivory (see Experimental Design), while grazing continued at the landscape level throughout the study period.

### 2.2 Experimental Design

At each site, five paired open‐top chambers (OTCs) and fenced control plots were established in October 2020. Each pair was spaced at least 10 m from the nearest neighboring pair. The cone‐shaped OTCs were constructed from extruded polycarbonate (86% transmission of visible and UV light; < 5% infrared transmission) and measured 0.6 m in height, with a basal diameter of 0.90 m and a top diameter of 0.45 m. Because sites are subject to summer grazing, sheep‐exclusion fences were installed around control plots, while the OTC structure itself prevented herbivory in warming plots. In total, the design comprised 50 experimental units (5 sites × 2 warming treatments × 5 plots). The spatial arrangement of OTCs and control plots was carefully planned to minimize potential interference effects, such as wind or snow shielding.

Temperature was monitored continuously in OTC and control plots over 36 months (October 2020–September 2023). At each site, one datalogger was installed inside an OTC and one in a control plot, yielding two continuous temperature records per site. HOBO MX23014 dataloggers were installed in October 2020, but they were replaced in July 2021 with TMS‐4 dataloggers (TOMST, Czech Republic; Wild et al. 2019) due to technical failures that resulted in partial data loss. HOBO MX23014 measured air temperature at 15‐cm height every 2 h. TMS‐4 sensors recorded air temperature at 15‐ and 2‐cm heights, and soil temperature and volumetric water content at 6‐cm depth, at 15‐min intervals. For air temperature, we used only measurements from the TMS‐4 sensor at 15‐cm height to ensure comparability with the HOBO data. Soil moisture was calculated using the standard calibration curve for peat soils (Wild et al. 2019).

All temperature data were aggregated to daily mean values prior to analysis. Growing degree days (GDD) were calculated by summing, across the growing season, the daily mean temperature exceeding a base threshold of 5°C (Körner 2003). Freezing degree days (FDD) were calculated as the cumulative sum of daily mean temperatures below 0°C. The meteorological growing season (*sensu* Körner et al. 2023) was defined from March to July.

### 2.3 Plant Sampling and Processing

Plant community composition and plant biomass were assessed in all experimental units at the peak of the growing season in early July for 3 years (2021–2023). All plants growing inside OTCs and control plots were identified to species level, except for some *Festuca* specimens that could only be identified at the genus level and were treated as distinct morphospecies. Plant species were further classified by growth form (Table S1). Species cover (%) was estimated visually within each plot and used as a proxy for plant abundance to calculate Shannon diversity, Pielou's evenness, and Inverse Simpson, implemented in the *vegan* package in R (Magurran 2004; Oksanen et al. 2022).

Species turnover between treatments was quantified using the Jaccard‐based beta‐diversity partitioning framework implemented in the R package betapart (Baselga and Orme 2012). Analyses were conducted at two levels: (1) within each year, pooling data across all sites, and (2) within each site, pooling data across years (see Supporting Information Appendix S3 for details). Community dissimilarity between Control and Warming plots was partitioned into total dissimilarity (β_jac), turnover (β_jtu; species replacement), and nestedness‐resultant dissimilarity (β_jne; species loss or gain).

Plant biomass, used as a proxy for plant growth, was estimated with the Robel pole method (Toledo et al. 2008). This method is widely used to measure structural attributes of vegetation, particularly in grasslands and shrublands, since it is simple and non‐destructive, allowing repeated measurements and avoiding disturbing the studied ecosystem. Briefly, plant height was measured at five random points per plot using a 1‐m pole marked in 10‐cm increments, with observers recording the point where vegetation obscured the pole from 4 m away. Plant biomass was estimated from site‐specific height–biomass regressions calibrated via destructive harvests of 0.5‐m^2^ vegetation samples, oven‐dried at 60°C for ≥ 48 h, and weighed. Biomass was expressed in g/m^2^.

Green leaves of *Nardus stricta* were collected from all experimental units in July 2022 for chemical analyses. In each OTC and control plot, leaves were collected from five randomly selected plants and stored in paper envelopes to be subsequently dried at 60°C for 48 h. Dried leaf samples were finely ground to a fine powder with a Retsch ball mill, and pooled per experimental unit, yielding 50 composite samples (5 sites × 2 treatments × 5 replicates). All chemical analyses were performed on these composite samples. The concentration of carbon (C) and nitrogen (N) was analyzed using a CN 802 Carbon Nitrogen Elemental Analyzer (VELP Scientifica, Italy). 100 mg of the ground leaf material was dried overnight at 110°C before analysis. Carbon was measured with an NDIR detector, and nitrogen was measured with a thermal conductivity detector. Carbon and nitrogen concentrations were expressed as % of dry leaf weight, and the C:N ratio was calculated as a mass ratio. Each sample was analyzed in triplicate to ensure reproducibility and accuracy. Phosphorus content was determined by digestion with aqua regia (HNO_3_: HCl, 1:3) at Laboratório de Química Agrícola, ISA (Lisbon, Portugal). Approximately 0.5 g of dried material was digested in 10 mL of aqua regia, filtered, and diluted to 50 mL with ultrapure water (adapted from European Standard EN13650). Phosphorus content was measured by ICP‐OES on an iCAP 7000 series spectrophotometer (Thermo Fisher Scientific, USA). Results were expressed as grams per kilogram of dry leaf weight (P; mg kg^−1^). Blanks, certified phosphorus standards, and spike recovery tests were included for quality control.

### 2.4 Data Analysis

All analyses were performed in **R version 4.4.1** (R Core Team 2024). Data processing and visualization were conducted using **dplyr, tidyr**, and **ggplot2** (Wickham et al. 2019). Data for the CUM site in 2023 were excluded due to extensive damage to the Open Top Chambers (OTCs) and monitoring equipment.

To account for the lack of spatial sensor replication within treatments, inference for daily microclimatic variables relied on highly frequent temporal replication. Consequently, temporal autocorrelation was explicitly modeled for daily time series of mean air temperature and soil moisture within each site × treatment combination (Figure S1). Mean daily air temperature (MeanT) was analyzed using generalized least squares (GLS) models based on approximately 10,250 daily observations across sites, treatments, and years. Fixed effects included site, treatment (warming vs. control), mean‐centered year (year_c), and their interactions. Seasonal variation was accounted for using harmonic terms (sine and cosine of day of year). Temporal autocorrelation inherent to daily climatic data was modeled using a first‐order autoregressive structure (AR1) within each site × treatment time series. Heteroscedasticity among sites was accommodated using a site‐specific variance structure. Models were fitted using restricted maximum likelihood (REML). Full model specifications and diagnostics are provided in the Supporting Information (Appendix S2).

Soil moisture (daily volumetric water content, VWC, %) was analyzed using GLS models analogous to those used for MeanT. Fixed effects included site, treatment, their interaction (site × treatment), and mean‐centered year. Because a single soil moisture sensor was installed per site × treatment combination, daily observations represent repeated measurements within each sensor time series. Temporal autocorrelation inherent to these measurements was therefore accounted for using a first‐order autoregressive (AR1) correlation structure. Site‐specific residual variances were also modeled to accommodate heteroscedasticity across study locations. Additional interaction terms involving year were tested but were not retained when they did not improve model fit.

Growing Degree Days (GDD) and Freezing Degree Days (FDD) were calculated for the meteorological growing season (March–July) and analyzed using one aggregated value per site × treatment × year combination (*n* = 28; CUM 2023 excluded due to infrastructure loss). Because these variables represent seasonal aggregates rather than daily time series, temporal autocorrelation structures were not required. GDD was analyzed using a Gamma generalized linear model with log link, and FDD using a Tweedie model with log link to account for zero values. Fixed effects included site, treatment, and year; interaction terms were excluded due to limited sample size and lack of improvement in model fit.

The effects of experimental warming on plant biomass, diversity, and leaf traits were analyzed using linear mixed‐effects models (LMMs) or linear models (LMs), depending on the response variable and sampling design. For biomass and diversity indices measured repeatedly in the same plots across years, LMMs included treatment, site, and year as fixed effects. To account for the hierarchical structure of the experiment and repeated measurements through time, plot identity was included as a random intercept nested within site (1 | site/plot). Models were fitted using restricted maximum likelihood (REML). For diversity metrics, interaction terms were initially tested but removed when non‐significant and when they did not improve model fit. For biomass, treatment × site and treatment × year interactions were initially tested to evaluate spatial and temporal heterogeneity in warming responses. However, the treatment × year interaction was not significant (*p* = 0.18) and did not improve model fit, and was therefore excluded from the final model. The three‐way interaction (treatment × site × year) was not fitted to avoid over‐parameterization and rank deficiency resulting from incomplete site–year replication. Inverse Simpson diversity and plant biomass were log_10_‐transformed before analysis to improve normality and homoscedasticity of residuals.

To evaluate the effects of experimental warming on *Nardus stricta* leaf nutrient content (N, C, C:N, and P), measured once in 2022, separate models were fitted for each response variable. All models included treatment, site, and their interaction (treatment × site) as fixed effects. Leaf N, C:N ratio, and P were analyzed using linear models with Gaussian error distributions, whereas leaf C was analyzed using a generalized linear model with a Gamma error distribution and log link, reflecting its strictly positive and right‐skewed distribution. To quantify uncertainty in the estimated percentage change in leaf nitrogen concentration under warming, 95% confidence intervals were calculated using non‐parametric bootstrap resampling (1000 iterations).

Linear mixed‐effects models were fitted using lme4, GLS models using nlme, and Tweedie models using glmmTMB (Bates et al. 2015; Pinheiro et al. 2025; Brooks et al. 2017). The significance of fixed effects was assessed using Type III ANOVA with Satterthwaite approximation implemented in lmerTest. Variance components of random effects were extracted using VarCorr, and marginal and conditional R^2^ values were calculated using MuMIn (Bartoń 2024). Model validation was performed through residual diagnostics. Assumptions of GLS models were evaluated using autocorrelation (ACF) and Q–Q plots, and additional model diagnostics were performed using the DHARMa package (Hartig 2024). Model selection was based on Akaike's Information Criterion (AIC), residual diagnostics, and overall model fit. Treatment effects were quantified as contrasts between Warming and Control using estimated marginal means calculated with emmeans (Lenth 2025). We report model‐based estimates with 95% confidence intervals derived from the fitted models. For Inverse Simpson diversity and biomass, which were log_10_‐transformed before analysis, contrasts were calculated on the log scale and back‐transformed to express percentage changes.

To summarize the magnitude of ecological responses to experimental warming, we calculated standardized effect sizes (Hedges' g) for plant species richness and aboveground biomass (Hedges and Olkin 1985; Nakagawa and Cuthill 2007). Effect sizes were computed separately for each year using site‐level means as the unit of replication (*n* = 5 sites per year; *n* = 4 in 2023 due to the loss of CUM). Treatment differences between Control and Warming were standardized by the pooled standard deviation among sites, and 95% confidence intervals were derived from the sampling variance of Hedges' g. Species‐specific responses were evaluated using raw differences in mean cover (Δ = Warming − Control) within each site × year combination and classified as Increased (Δ > 0), Decreased (Δ < 0), Gained (present only under warming), or Lost (present only under control). Standardized effect sizes (Hedges' g) were calculated for species with sufficient replication using plot‐level cover data within each site × year combination, requiring occurrence in both treatments and non‐zero variance in at least one treatment group. *Nardus stricta* occurred in all sites, plots, and sampling years. For this species, effect sizes were first calculated separately for each site × year combination and then combined to obtain a site‐level estimate using an inverse‐variance weighted mean (weight = 1/Var (g)), with corresponding 95% confidence intervals. For spatially restricted species or cases with insufficient replication, responses were summarized descriptively using Δ values. Effect sizes were interpreted following conventional thresholds: very small (|g| < 0.2), small (0.2 ≤ |g| < 0.5), medium (0.5 ≤ |g| < 0.8), large (0.8 ≤ |g| < 1.2), very large (1.2 ≤ |g| < 2.0), and huge (|g| ≥ 2.0). Detailed formulas are provided in Supporting Information (Appendix E in Appendix S3).

## 3 Results

### 3.1 Effect of Warming on Micro‐Environmental Characteristics

**FIGURE 2:**
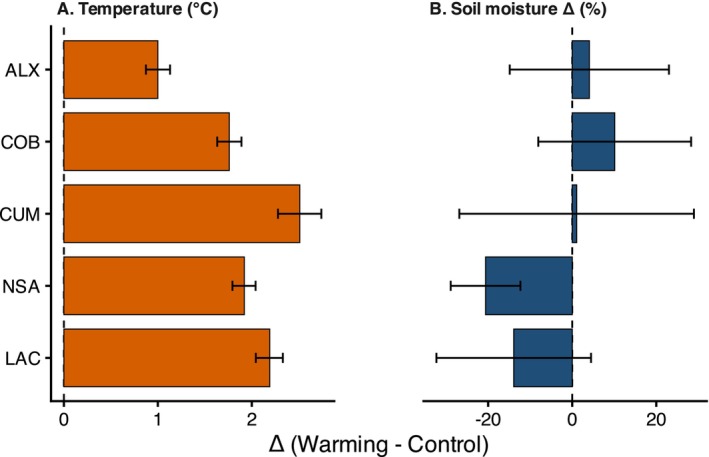
Estimated warming effects on (A) daily mean air temperature (MeanT) and (B) soil volumetric water content (VWC) across study sites. Bars represent model‐estimated contrasts between warming and control treatments (Δ = Warming − Control) derived from generalized least squares (GLS) models, with 95% confidence intervals. For VWC, values are expressed as percentage change relative to control conditions. The dashed vertical line indicates no treatment effect (Δ = 0). Confidence intervals that do not overlap zero indicate statistically supported treatment effects at α = 0.05. Variables are shown on different x‐axis scales.

The open‐top chambers (OTCs) significantly increased mean air temperature across sites, confirming the effectiveness of the experimental warming treatment (Figure 2A). Across sites and years, OTCs increased mean air temperature by approximately 1.9°C ± 0.19°C on average, and by 2.61°C ± 0.38°C during the growing season. The magnitude of warming differed among sites (site × treatment interaction: *p* = 0.02; Table S2; Figure 2A), ranging from **~**1.0°C at ALX (1823 m) to ~2.5°C at CUM (1875 m), with intermediate increases at COB (~1.76°C), NSA (~1.92°C), and LAC (~2.19°C). No overall temporal trend in the warming effect was detected (year effect: *p* = 0.09), although interannual variation differed among sites (site × year interaction: *p* < 0.001; Figure S1b). Overall, the OTC treatment produced a consistent increase in air temperature while retaining natural spatial and interannual variability.

Experimental warming significantly increased growing degree days (GDD) and reduced freezing degree days (FDD) during the growing season (Figure S2; Table S3). On average, GDD increased from 823 ± 29.9 degree‐days under control conditions to 1116 ± 53.6 under warming (mean ± SE), corresponding to a 35% increase (χ^2^ = 39.15, *p* < 0.0001). In contrast, FDD decreased from 6.36 ± 2.22 to 1.14 ± 0.42 degree‐days, representing an 82% reduction (χ^2^ = 16.43, *p* < 0.0001). Across sites, GDD increased from 37% (ALX) to ~50% (CUM), while freezing exposure declined 80–100% at all sites, with a minimum value (65%) in ALX. FDD varied significantly among years (χ^2^ = 14.32, *p* < 0.001), whereas GDD showed no overall year effect (χ^2^ = 1.33, *p* = 0.249). Importantly, the direction and magnitude of warming effects on both metrics were consistent across years.

The effect of warming on soil volumetric water content (VWC) differed among sites (Figure 2B, Tables S4 and S5), with significant effects of site (F_4,6696_ = 4.59, *p* = 0.0011), treatment (F_1,6696_ = 14.72, *p* = 0.0001), and their interaction (F_4,6696_ = 3.54, *p* = 0.0069). Warming significantly reduced soil moisture at NSA (Δ = −0.206 ± 0.042 SE, *p* < 0.001). Responses at the other sites were weak or non‐significant, including a modest but non‐significant decrease at LAC, while no significant treatment effects were detected at ALX, COB, or CUM. Soil moisture did not vary significantly with year (year_c: F_1,6696_ = 0.84, *p* = 0.36), indicating no consistent temporal trend across the study period.

### 3.2 Effect of Warming on Plant Diversity, Growth, and Nutrient Content

A total of 36 plant species were recorded across sites and years, the majority being perennial forbs or grasses, primarily from families such as Poaceae, Caryophyllaceae, Fabaceae, and Ericaceae (Table S1). *Nardus stricta* was the dominant species across all sites and in all years, accounting for a mean cover of 65%. Iberian endemic taxa, including *Festuca rothmaleri* and *Galium saxatile* var. *vivianum*, together with *Festuca henriquesii*, which is restricted to Serra da Estrela, were recorded within experimental plots. At COB, one of the highest elevation sites, *F. henriquesii* reached up to 38% cover. Three moss taxa, *Aulacomnium palustre* , *Orthotrichum stramineum*, and *Sphagnum* spp., were recorded in the wetter grasslands.

**FIGURE 3:**
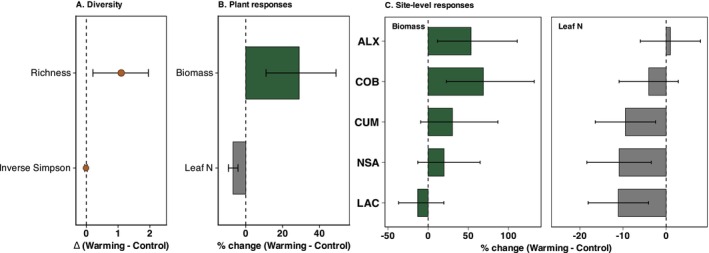
Effects of experimental warming on plant diversity and functional responses. (A) Global model‐estimated contrasts (Δ = Warming − Control) for species richness and inverse Simpson diversity. (B) Model‐estimated percentage change under warming for aboveground biomass and leaf nitrogen concentration. (C) Site‐level model‐estimated percentage change under warming for aboveground biomass and leaf nitrogen concentration. Points and bars represent estimated marginal means derived from the fitted models, with horizontal lines indicating 95% confidence intervals (CI). The vertical dashed line denotes no treatment effect (Δ = 0 in panel A; 0% change in panels B and C).

Species richness was significantly higher under warming (F_1,45_ = 6.24, *p* = 0.016; Figure 3, Tables S6 and S7), increasing by 1.08 species on average (~22% relative to control). Richness also differed significantly among sites (F_4_,_44.8_ = 8.31, *p* < 0.001), but not among years (*p* = 0.285). The positive warming effect was observed across all sites, with increases ranging from +0.73 species at NSA to +2.00 species at ALX. The magnitude of this response was moderate and relatively similar across years (Hedges' g = 0.52–0.87; Figure S3). In contrast, Shannon diversity, inverse Simpson diversity, and Pielou's evenness were not significantly affected by warming (all *p* > 0.05), although evenness showed a marginal tendency to decline (*p* = 0.061). These diversity indices varied significantly among sites (*p* < 0.001) and years (*p* < 0.0001; Table S7), with 2022 consistently showing lower diversity values than 2021 and 2023. Across sites, LAC and CUM supported the highest richness and Shannon diversity, COB showed the highest evenness and dominance‐weighted diversity, whereas NSA consistently exhibited the lowest diversity values (Table S6).

Aboveground biomass was also higher under warming (168.61 ± 10.40 SE g m^−2^) than under control conditions (129.38 ± 7.25 SE g m^−2^; Tables S8 and S9). Experimental warming had a significant overall effect on biomass (F_1_,41.5 = 12.06, *p* = 0.001), corresponding to an average increase of approximately 29% (95% CI: 11%–49%). The magnitude of this response was moderate across years (Hedges' g = 0.59–0.85; Figure S3). However, the warming response varied among sites (treatment × site: F_4_,40.2 = 2.67, *p* = 0.046). Biomass increased significantly at ALX (+53%) and COB (+69%), whereas responses at CUM (+22%), NSA (+20%), and LAC (−13%) were weaker and not statistically significant (Figure 3C, Table S9). Biomass also differed strongly among years (F_2_,88.5 = 66.70, *p* < 0.001), with higher values in 2021 than in 2022 and 2023. However, the effect of warming did not vary among years (treatment × year: F_2_,86.4 = 1.75, *p* = 0.18).

Experimental warming reduced leaf N from 1.51% in control plots to 1.40% in warmed plots (Table S10), corresponding to an average decline of 6.9% (95% CI: −9.4% to−4.2%; Figure **3**B). This reduction was accompanied by a significant increase in the C:N ratio (estimate = 1.84, *p* < 0.05). Warming had no detectable effect on leaf carbon (C) or phosphorus (P). Although the treatment × site interaction was not significant, the magnitude of the reduction varied among sites, with stronger declines at CUM, LAC, and NSA and weaker responses at ALX and COB (Figure 3C). Significant differences among sites were also observed: leaf *P* was highest at COB and lowest at CUM, while the C:N ratio was highest at NSA and lowest at ALX (Table S10).

### 3.3 Plant Community Composition Diverged Between Warming and Control Plots

**FIGURE 4:**
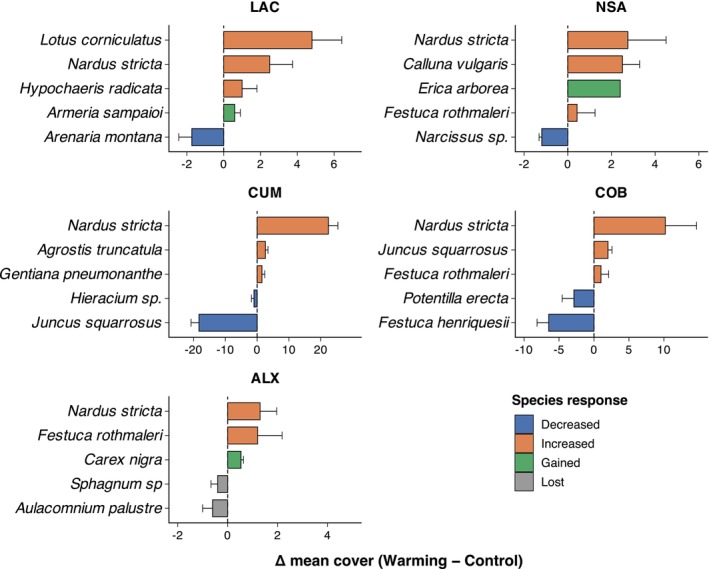
Plant species cover responses to experimental warming across sites. Bars show the mean differences in cover (Δ = Warming − Control) for each species within each site, calculated from site × year combinations and averaged across years. Positive values indicate higher cover under warming, whereas negative values indicate lower cover relative to control conditions. Error bars (± SE) are shown for species recorded in more than one year. Species are colored according to response category: Increased (Δ > 0), Decreased (Δ < 0), Gained (present only under warming), and Lost (present only in control plots). The vertical dashed line indicates no treatment effect (Δ = 0). Standardized effect sizes (Hedges' g) for species with sufficient replication are reported in Table S12.

Overall, Jaccard dissimilarity remained consistent across years (≈0.58–0.59; Table S11), whereas spatial variation among sites was more pronounced. Both species turnover and nestedness contributed similarly to total beta diversity (each ≈0.28–0.32 overall), although their relative importance varied among sites. Turnover contributed more strongly at LAC and COB, whereas nestedness was relatively more important at NSA (Table S11). Direct comparisons between control and warming plots revealed moderate species turnover (0.26–0.31), indicating persistent compositional shifts under warming. Although turnover magnitude was comparable among years, the identity of species driving these changes differed markedly among sites (Figure 4; Table S12), underscoring spatially heterogeneous responses.

These community‐level shifts were largely driven by changes in dominant and locally abundant species. At the species level, the dominant grass *Nardus stricta* increased by 14% on average under warming across the study system. However, this response was spatially heterogeneous: increases were strongest at CUM (+42%) and COB (+31%), whereas responses at ALX, LAC, and NSA were minimal (< 5%). Consistent with these patterns, site‐level effect sizes were largest at CUM (g > 2) and smaller at the remaining sites, indicating that warming amplified *N. stricta* dominance primarily where thermal accumulation was greatest (Figure 4, Table S12).

Several species exhibited contrasting site‐specific responses (Figure 4, Table S12). At COB, the endemic *Festuca henriquesii* declined consistently across all 3 years (mean−17%; mean g = −0.20), suggesting sensitivity to warming at this high‐elevation site. *Juncus squarrosus* showed strong spatial heterogeneity: it declined sharply at CUM (mean g = −1.32 across 2021–2022; −90% on average) but increased at ALX and COB (mean g≈0.40), highlighting contrasting local sensitivities. At LAC, warming favored *Lotus corniculatus* (mean g = 1.14 across 2 years), whereas *Arenaria montana* declined (mean g = −0.54). At NSA, shrub species such as *Calluna vulgaris* showed positive responses (mean g = 0.5), while other taxa exhibited weak or variable changes. The Iberian endemic *Festuca rothmaleri* showed a modest increase under warming at several sites, although its overall abundance remained low. Bryophytes were particularly sensitive at ALX, where *Aulacomnium palustre* and *Sphagnum* spp. occurred in control plots but were absent under warming across multiple years, suggesting local extirpation under elevated temperatures. Colonization events were also observed, with several species ( *Carex nigra* , *Aira praecox* , *Armeria sampaioi*, *Orthotrichum stramineum* ) occurring only under warming at specific sites.

## 4 Discussion

This study provides the first multi‐year experimental assessment of warming impacts on subalpine *Nardus stricta* grasslands in Mediterranean mountains. Experimental warming increased mean air temperature by +1.9°C on average, consistent with projected near‐term climate change scenarios (IPCC 2021). Warming led to increased plant biomass and species richness, shifts in community composition, and reduced foliar nitrogen concentrations in the dominant speci**es**. The magnitude of warming observed falls within the range reported for other OTC experiments in alpine and subalpine ecosystems (typically 1°C–3°C). However, the magnitude and direction of ecological responses varied among sites along the environmental gradient, highlighting the context‐dependent nature of ecosystem responses to warming. Our results also indicate the vulnerability of cold‐adapted and endemic taxa alongside the resilience of dominant species such as *N. stricta* , with potential implications for the conservation and management of these priority mountain grassland ecosystems.

Warming consistently resulted in significant increases in species richness and aboveground biomass, although the strength of these responses differed among sites, a result coherent with research in subalpine grasslands in temperate mountains (Peppler‐Lisbach et al. 2025 and references therein). In contrast, Shannon and inverse Simpson diversity were not significantly affected by warming, while Pielou's evenness showed a marginal tendency to decline (*p* = 0.061). Although species richness increased consistently across sites, the slight reduction in evenness suggests a strengthening of dominance structure under warming. The expansion of *Nardus stricta* provides a mechanistic explanation for the marginal decline in evenness and the stability of dominance‐weighted diversity indices under warming. Thus, warming promoted species additions without proportionally redistributing relative abundances, resulting in higher richness but limited change in overall diversity metrics. These findings underscore the differential sensitivity of biodiversity components to warming and highlight the interplay between species‐level responses and local abiotic conditions (Fei et al. 2017).

Biomass increased in response to enhanced thermal accumulation and reduced freezing exposure, although this response was weaker at sites with lower baseline soil water availability. Temperature commonly limits plant growth at high elevations; accordingly, warming—particularly winter warming that extends the growing season—has been shown to enhance primary productivity in cold temperate systems (Kreyling et al. 2019; Rustad et al. 2001). In Mediterranean mountains, however, this positive effect may be constrained by limited summer water availability, which can intensify under warming (Guiot and Cramer 2016; Nogués‐Bravo et al. 2008; Sebastià et al. 2008). Consistent with this framework, LAC, characterized by the lowest baseline soil moisture, exhibited the weakest biomass response, suggesting stronger water limitation. These patterns underscore the role of soil moisture as a key moderator of warming effects (Bjorkman et al. 2018; Peng et al. 2020; Vandvik et al. 2020).

Although warming generated a consistent thermal signal across sites, ecological outcomes were context‐dependent: compositional restructuring was most pronounced at the highest‐elevation site, whereas productivity responses were constrained at the driest site, indicating joint control by thermal release and water limitation. In addition, despite the observed increase in plant biomass under warming, foliar nitrogen concentration in *N. stricta* was significantly lower in warmed plots. This reduction may reflect a dilution effect associated with accelerated growth and/or warming‐induced shifts in soil nutrient cycling (Sardans et al. 2017). Lower leaf nitrogen may have important functional and management implications, since high‐elevation grasslands dominated by *N. stricta* are traditionally used for summer grazing by cows and sheep (Korzeniak 2016; Monteiro et al. 2020). Despite the generally low protein content and digestibility reported for *N. stricta* leaves, this species represents an important food resource due to its high abundance in these pasturelands (Bovolenta et al. 2008). Consequently, reductions in foliar nitrogen under warming may influence forage quality, as nitrogen is closely linked to protein content in herbivore diets (Soussana and Lüscher 2007; Bovolenta et al. 2008), potentially affecting livestock nutrition and associated ecological and socio‐economic dynamics.

Changes in species composition and community diversity under warming are expected, as generalist thermophilic species from lower elevations expand their distribution, while specialized species from higher elevations decline (i.e., Gottfried et al. 2012; Lamprecht et al. 2018; Pauli et al. 2012). In our study, beta diversity analyses revealed that both species replacement (turnover) and nestedness (species loss or gain without replacement) contributed similarly to overall community differentiation, with their relative importance varying slightly among years and sites. Thus, warming‐induced community changes proceed through a combination of species replacement and species loss/gain processes depending on local conditions and temporal context. Consistent with these patterns, delta values and standardized effect sizes indicated differential directional shifts in species cover under warming, particularly among rare or patchily distributed taxa.

As the most abundant species across all five sites, *N. stricta* showed the largest increase in cover, rising by 6.5 percentage points on average (a 14% relative increase) across site × year combinations—from 61.9% in control plots to 68.4% in warmed plots—reinforcing its role as a dominant and resilient component of these subalpine grasslands. The magnitude of this response varied among sites, with particularly strong increases at the highest‐elevation site. Such expansion may reduce local heterogeneity, as the dense tussock growth form and extensive root system of *N. stricta* can competitively suppress neighboring species (Kurtogullari et al. 2020; Sebastià 2004). Responses of other plant species were likewise strongly site‐dependent. Despite the current broader shrub encroachment trend in mountain grasslands (Gómez‐García et al. 2023), shrub responses to warming in our study were localized rather than system‐wide, being significant in one of the sites located at a lower altitude (higher mean temperature) and with a significant reduction in soil moisture. Warming also led to the appearance of *Lotus corniculatus* , a leguminous species known for its tolerance to summer drought, in the warmest and driest site. In contrast, the snow‐associated endemic *Festuca henriquesii*, restricted to one of the high‐elevation sites, declined consistently under warming (mean−17% across years). Similarly, *Juncus squarrosus*, a diagnostic species for these grasslands (Chytrý et al. 2020), exhibited a strong decline at the highest site, while moisture‐dependent mosses showed site‐specific responses. Overall, warming amplified existing environmental gradients rather than producing uniform compositional shifts, with cold‐ and moisture‐associated taxa showing greater sensitivity in thermally responsive or water‐limited contexts. Such responses highlight the vulnerability of endemic and specialist species and reinforce the context‐dependent nature of vegetation change under climate warming (Klanderud 2008; Peng et al. 2020).

## 5 Conclusions and Implications for Conservation

Experimental warming increased plant biomass and species richness, altered community composition, and reduced foliar nitrogen concentration in the dominant grass species in high‐elevation sub‐Mediterranean grasslands of southern Europe. The decline of endemic and cold‐adapted species, together with the expansion of dominant taxa, suggests emerging biotic homogenization and functional turnover that may ultimately alter both the conservation value and ecological functioning of these grasslands. The consequences of these shifts for overall forage quality also require further investigation. Our findings indicate that vegetation responses to warming were strongly site‐dependent. Plant biomass responses were primarily associated with baseline soil water availability, whereas species turnover and individual plant responses were more closely linked to baseline temperature conditions. These findings highlight the need to explicitly incorporate climate resilience into conservation strategies for critically endangered Mediterranean mountain habitats. Management approaches that enhance ecosystem resilience—such as maintaining traditional grazing regimes, identifying and protecting mesic microhabitats, or considering assisted migration for particularly sensitive taxa—may help mitigate the decline of cold‐adapted and endemic species while sustaining ecosystem functioning. Preserving environmental heterogeneity and climate refugia may therefore help buffer these mountain grasslands against warming‐driven biodiversity loss.

## Author Contributions

**Marta Correia:** conceptualization (supporting), data curation (equal), formal analysis (equal), investigation (equal), methodology (equal), writing – original draft (lead), writing – review and editing (lead). **Alexandre Silva:** data curation (lead), formal analysis (supporting), investigation (supporting), writing – review and editing (supporting). **Susana Rodríguez‐Echeverría:** conceptualization (lead), data curation (equal), formal analysis (equal), funding acquisition (lead), investigation (equal), methodology (equal), project administration (lead), writing – original draft (supporting), writing – review and editing (equal).

## Funding

This work was developed within the research project PTDC/BIA‐CBI/30215/2017 (https://doi.org/10.54499/PTDC/BIA‐CBI/30215/2017) funded by the European Union (Portugal 2020—Program CENTRO‐01‐0145‐FEDER‐030215) and the FCT—Fundação para a Ciência e Tecnologia, I.P. We also had the support of FCT—Fundação para a Ciência e Tecnologia, I.P., in the framework of the Project UID/04004/2025—Centre for Functional Ecology—Science for the People and the Planet, with DOI identifier 10.54499/UID/04004/2025 (https://doi.org/10.54499/UID/04004/2025).

## Conflicts of Interest

The authors declare no conflicts of interest.

## Supporting information


**Table S1:** List of plant species recorded in this study. Species endemic to the Iberian Peninsula are highlighted in light green, and species endemic to Serra da Estrela are shown in bold green.
**Table S2:** Results of generalized least squares (GLS) models testing spatial and temporal variation in the experimental warming effect on daily mean air temperature. The response variable represents the daily temperature difference between warming and control plots (ΔT = Warming—Control). Temporal autocorrelation within each site time series was modeled using a first‐order autoregressive correlation structure (AR (1)), and heteroscedasticity among sites was accounted for using a site‐specific variance structure (varIdent). Year was mean‐centered (year_c) to facilitate interpretation of model intercepts. Seasonal variation was modeled using sinusoidal terms (sin1 and cos1). (A) Fixed‐effect parameter estimates of the GLS model; (B) F‐tests evaluating the effects of site, year (year_c), seasonal components, and their interaction; (C) Site‐specific warming effects (ΔT = Warming—Control) estimated using estimated marginal means (emmeans). Values represent the warming effect at the mean year of the study (year_c = 0). Standard errors (SE), 95% confidence intervals, and *p*‐values testing whether ΔT differs from zero are shown.
**Table S3:** Effects of experimental warming, site, and year on growing degree days (GDD) and freezing degree days (FDD). Results of generalized linear models testing the effects of treatment, site, and year on thermal accumulation and freezing exposure accumulated between March and July. GDD was analyzed using a Gamma GLM (log link), and FDD using a Tweedie GLMM (log link; glmmTMB). Model explanatory power was high for GDD (Nagelkerke R^2^ = 0.74) and moderate for FDD (McFadden R^2^ = 0.25). The table includes (a) analysis of deviance tables (Type II Wald χ^2^ tests), (b) observed site‐specific means and ratios (Warming/Control) with confidence intervals and *p*‐values, and (c) pairwise comparisons of marginal means among years (emmeans, Tukey‐adjusted).
**Table S4:** Mean daily ± SE soil volumetric water content % (VWC ± SE) for each site × year × treatment combination (*n* indicates the number of daily sensor observations per group).
**Table S5:** Results of the generalized least squares (GLS) model testing the effects of site, treatment, and their interaction on daily soil moisture (volumetric water content, VWC%), with year included as a mean‐centered continuous covariate (year_c). Soil moisture was analyzed using 6672 daily observations across all site × treatment combinations. Temporal autocorrelation in daily sensor measurements was accounted for using a first‐order autoregressive correlation structure (AR1), and heteroscedasticity among sites was accommodated using a site‐specific variance structure (varIdent). The table includes: (A) Type III ANOVA results for fixed effects in the GLS model. (B) Estimated warming effects on soil moisture for each site (ΔVWC = Warming − Control). Values are expressed as percentage differences derived from estimated marginal means of the GLS model.
**Table S6:**. Mean (± SE) plant diversity indices (species richness, Shannon diversity, inverse Simpson diversity, and Pielou evenness) for each site × treatment combination. Values are calculated from plot‐level data.
**Table S7:**. Results of linear mixed‐effects models (LMMs) for plant diversity indices. Models included site, year, and treatment as fixed effects and plot as a random intercept. The site × treatment interaction was tested but excluded from the final models because it was not significant and did not improve model fit. Specifically, the interaction between warming treatment and site was not significant for species richness (F_4_,40.5 = 0.28, *p* = 0.891) or inverse Simpson diversity (F_4_,42.5 = 1.54, *p* = 0.209), indicating that warming effects on diversity were consistent across sites. The table includes: (A) Type III ANOVA results (df, F‐values, and *p*‐values) for all diversity indices; marginal and conditional R^2^ and variance components of the random intercept (plot) are also reported for each model. (B) Estimated marginal means and treatment contrasts for species richness. (C) Tukey‐adjusted pairwise comparisons among sites. (D) Pairwise comparisons among years estimated using *emmeans*.
**Table S8:** Mean aboveground plant biomass (g m^−2^) per site, year and treatment. Values represent mean ± SE (*n* = 5 plots per treatment).
**Table S9:** Linear mixed‐effects model testing the effects of experimental warming on log_10_‐transformed plant biomass. The model included treatment, site, year, and the treatment × site interaction as fixed effects, with plot included as a random intercept. An interaction between treatment and year was initially tested but was not significant (*p* = 0.18) and did not improve model fit; therefore, it was excluded from the final model. (A) Type III ANOVA table (Satterthwaite approximation) for fixed effects. (B) Estimated treatment effects within each site (emmeans; Kenward–Roger degrees of freedom). (C) Pairwise comparisons between years (emmeans contrasts; Tukey‐adjusted *p*‐values).
**Table S10:** Summary of linear (LM) and generalized linear (GLM) models assessing the effects of the warming treatment (OTC), site, and their interaction on *Nardus stricta* leaf nutrient concentrations (%) measured at the end of the 2022 growing season. The carbon (C) model was fitted using a Gamma GLM with a log link. Marginal R^2^ values represent the proportion of variance explained by fixed effects. Estimated marginal means for the C model are presented on the response scale. (A) Type II/III ANOVA results for nutrient models (N, C, C:N, and *P*) (B1). Estimated marginal means of leaf N and C:N by treatment (B2). Estimated marginal means of leaf N, C, C:N, and *P* by site (averaged across treatments).
**Table S11:** Beta diversity based on Jaccard dissimilarity between control and warming plots within each year (A) and within each site and year (B). Total dissimilarity (β.jac) is split into turnover (β.jtu: species replacement) and nestedness (β.jne: species loss/gain without replacement). Higher values indicate greater compositional differences between treatments.
**Table S12:** Site‐specific responses (Δ cover) and standardized effect sizes (Hedges' g) for dominant plant species *Nardus stricta* and other non‐dominant species with sufficient replication to estimate variance. Hedges' g represents the bias‐corrected standardized mean difference between Warming and Control treatments, calculated separately for each site × year using plot‐level replication (*n* = 5 plots per treatment). For each species, *g* mean represents the mean effect size across years. Effect sizes were calculated only when species (i) occurred in both treatments within a site × year combination, (ii) showed non‐zero variance in at least one treatment group, and (iii) allowed estimation of the pooled standard deviation. Species present in fewer years are included when effect sizes can be estimated, but should be interpreted cautiously due to lower temporal replication.
**Figure S1:** (a) Diagnostic plots of the GLS model used to estimate warming effects on daily temperature differences (ΔT). Panels show (A) autocorrelation function (ACF) of normalized residuals, (B) residuals versus fitted values, and (C) normal Q–Q plot. Residual diagnostics indicate no strong remaining temporal autocorrelation and approximately normally distributed residuals. (b) Site‐ and year‐specific effects of experimental warming (Δ Warming − Control). Annual estimates of the warming effect on mean air temperature (Δ MeanT, °C; Warming − Control) across study sites. Points represent model estimates derived from a generalized least squares model accounting for temporal autocorrelation (AR1) and seasonal variation (sine and cosine terms). Error bars indicate 95% confidence intervals. The horizontal dashed line denotes no warming effect (Δ = 0). Panels correspond to the five study sites (NSA, LAC, COB, ALX, and CUM). Estimates are shown for the years 2020–2023, except for CUM in 2023, where data were unavailable.
**Figure S2:** Seasonal warming effects on Growing Degree Days (GDD) and Freezing Degree Days (FDD) computed for the meteorological growing season (March–July). Bars show Δ (warming − control) in cumulative degree‐days (°C·days) for each site and year. Positive values indicate higher values under warming, whereas negative values indicate lower values compared to control conditions.
**Figure S3:** Standardized effect sizes (Hedges' g±95% CI) describing the magnitude of warming effects on plant species richness and aboveground biomass for each study year. Effect sizes were calculated using site‐level means as replicates (*n* = 5 sites per year; *n* = 4 in 2023 after the loss of CUM). Positive values indicate higher values under warming relative to control.

## Data Availability

All datasets are archived and publicly available at DRYAD under the link: https://datadryad.org/dataset/doi:10.5061/dryad.sf7m0cgnt. The repository is currently private and will be made publicly accessible upon publication. Additional related data are provided as Supporting Information accompanying this manuscript.
