# Correction to “Warming Leads to Biomass Increase, Leaf Nitrogen Decline, and Community Turnover in Mediterranean *Nardus stricta* Grasslands”

**DOI:** 10.1002/ece3.74464

**Published:** 2026-10-04

Correia, M. , A. Silva , and S. Rodríguez‐Echeverría . 2026. “Warming Leads to Biomass Increase, Leaf Nitrogen Decline, and Community Turnover in Mediterranean *Nardus stricta* Grasslands.” Ecology and Evolution 16, no. 4: e73537. 10.1002/ece3.73537.PMC1310097242028424

In the Funding section, the funding source “PRESINMED—BiodivNBS/0012/2023 (https://sciproj.ptcris.pt/176382PRJ), funded by Biodiversa+NBS 2023” was missing. The updated Funding section is as follows:

## Funding

This work was developed within the research project PTDC/BIA‐CBI/30215/2017 (https://doi.org/10.54499/PTDC/BIA‐CBI/30215/2017) funded by the European Union (Portugal 2020—Program CENTRO‐01‐0145‐FEDER‐030215) and the FCT—Fundação para a Ciência e Tecnologia, I.P. We also had the support of FCT—Fundação para a Ciência e Tecnologia, I.P., in the framework of projects PRESINMED—BiodivNBS/0012/2023 (https://sciproj.ptcris.pt/176382PRJ), funded by Biodiversa+NBS 2023 and UID/04004/2025—Centre for Functional Ecology—Science for the People and the Planet, with DOI identifier 10.54499/UID/04004/2025 (https://doi.org/10.54499/UID/04004/2025).

We apologize for this error.

