# Supplementary material for: Warming Leads to Biomass Increase, Leaf Nitrogen Decline, and Community Turnover in Mediterranean Nardus stricta Grasslands
Source: Ecol Evol. 2026 Apr 22;16(4):e73537. doi: 10.1002/ece3.73537 (PMC13100972; doi:10.1002/ece3.73537)
Supplement: Supplementary file 1 — Table S1: List of plant species recorded in this study. Species endemic to the Iberian Peninsula are highlighted in light green, and species endemic to Serra da Estrela are shown in bold green. Table S2: Results of generalized least squares (GLS) models testing spatial and temporal variation in the experimental warming effect on daily mean air temperature. The response variable represents the daily temperature difference between warming and control plots (ΔT = Warming—Control). Temporal autocorrelation within each site time series was modeled using a first‐order autoregressive correlation structure (AR (1)), and heteroscedasticity among sites was accounted for using a site‐specific variance structure (varIdent). Year was mean‐centered (year_c) to facilitate interpretation of model intercepts. Seasonal variation was modeled using sinusoidal terms (sin1 and cos1). (A) Fixed‐effect parameter estimates of the GLS model; (B) F‐tests evaluating the effects of site, year (year_c), seasonal components, and their interaction; (C) Site‐specific warming effects (ΔT = Warming—Control) estimated using estimated marginal means (emmeans). Values represent the warming effect at the mean year of the study (year_c = 0). Standard errors (SE), 95% confidence intervals, and p‐values testing whether ΔT differs from zero are shown. Table S3: Effects of experimental warming, site, and year on growing degree days (GDD) and freezing degree days (FDD). Results of generalized linear models testing the effects of treatment, site, and year on thermal accumulation and freezing exposure accumulated between March and July. GDD was analyzed using a Gamma GLM (log link), and FDD using a Tweedie GLMM (log link; glmmTMB). Model explanatory power was high for GDD (Nagelkerke R2 = 0.74) and moderate for FDD (McFadden R2 = 0.25). The table includes (a) analysis of deviance tables (Type II Wald χ2 tests), (b) observed site‐specific means and ratios (Warming/Control) with confidence intervals and p‐values, [file ECE3-16-e73537-s001.docx]

**Supporting information**

Warming leads to biomass increase, leaf nitrogen decline, and community turnover in Mediterranean *Nardus stricta* grasslands

1. **Plant species composition and soil characteristics of study sites**

**Table S1.** List of plant species recorded in this study. Species endemic to the Iberian Peninsula are highlighted in light green, and species endemic to Serra da Estrela are shown in bold green.

| **Species** | **Family** | **Growth form** |
| --- | --- | --- |
| *Aira praecox* L. | Poaceae | Annual grass |
| *Arenaria montana* L. | Caryophyllaceae | Perennial forb |
| *Armeria sampaioi* (Bernis) Nieto Fel. | Plumbaginaceae | Perennial forb |
| *Aulacomnium palustre* (Hedw.) Schwägr. | Aulacomniaceae | Moss |
| *Calluna vulgaris* (L.) Hull | Ericaceae | Subshrub |
| *Carex nigra* (L.) Reichard | Cyperaceae | Perennial hemicryptophyte, geophyte |
| *Cerastium ramosissimum* Boiss. | Caryophyllaceae | Perennial forb |
| *Corynephorus canescens* (L.) P.Beauv. | Poaceae | Perennial grass |
| *Erica arborea* L. | Ericaceae | Shrub |
| ***Festuca henriquesii* Hack*.*** | Poaceae | Perennial grass |
| *Festuca rothmaleri* (Litard.) Markgr.-Dann. | Poaceae | Perennial grass |
| *Festuca sp.1* | Poaceae | Grass |
| *Festuca sp.2* | Poaceae | Grass |
| *Festuca sp.3* | Poaceae | Grass |
| *Galium saxatile var. vivianum* (Kliphuis) Ortega Oliv. & Devesa | Rubiaceae | Perennial forb or subshrub |
| *Genista anglica* L. | Fabaceae | Shrub |
| *Gentiana pneumonanthe* L. | Gentianaceae | Perennial forb |
| *Hyeracium sp.* | Asteraceae | Perennial forb |
| *Hypochaeris radicata* L. | Asteraceae | Perennial forb |
| *Juncus squarrosus* L. | Juncaceae | Perennial hemicryptophyte |
| *Lotus corniculatus* L. | Fabaceae | Perennial forb |
| *Molineriella laevis* (Brot.) Rouy | Poaceae | Perennial grass |
| *Narcissus bulbocodium* L. | Amaryllidaceae | Bulbous geophyte |
| *Narcissus sp.* | Amaryllidaceae | Bulbous geophyte |
| *Nardus stricta* L. | Poaceae | Perennial grass |
| *Neoschischkinia truncatula* (Parl.) Valdés & H.Scholz | Poaceae | Perennial Grass |
| *Ornithopus perpusillus* L. | Fabaceae | Annual forb |
| *Orthotrichum stramineum* Hornsch. ex Brid. | Orthotrichaceae | Moss |
| *Pedicularis sylvatica* L. | Orobanchaceae | Hemiparasitic biennial or perennial forb |
| *Polygala serpyllifolia* Hosé | Polygalaceae | Perennial subshrub |
| *Potentilla erecta* (L.) Reichard | Rosaceae | Perennial forb |
| *Ranunculus bulbosus* L. | Ranunculaceae | Tuberous geophyte |
| *Rumex acetosella* L. | Polygonaceae | Perennial forb |
| *Spergula morisonii* Boreau | Caryophyllaceae | Forb |
| *Sphagnum sp.* | Sphagnaceae | Moss |
| *Teesdalia nudicaulis* (L.) W.T.Aiton | Brassicaceae | Annual forb |

**Appendix S1. Methods for soil chemical and texture analyses**

In November 2020, three soil samples were randomly collected from each control plot at each site and pooled into a composite sample for soil chemical analyses (n = 25). Soil pH, water content (SWC), organic matter (SOM), and nitrogen (N) were determined for each grassland. Soil water content was measured by oven-drying soil subsamples at 80 °C for 48 h. The remaining soil was air-dried and sieved through a 2-mm mesh prior to further analyses. Soil pH was measured in a 1:2.5 soil-to-distilled water suspension (LQARS, 1977; Rodríguez-Echeverría et al. 2009). Soil organic matter was estimated using the loss-on-ignition method by combustion at 450 °C for 4 h in a muffle furnace (Nelson & Sommers 1982). Soil nitrogen content was determined using a CN 802 Carbon Nitrogen Elemental Analyzer (VELP Scientifica, Italy). Each sample was analyzed in triplicate to ensure reproducibility.

Additionally, three soil samples were randomly collected from each study site (n = 15) to determine soil texture (granulometric composition). Particle-size distribution and the relative proportions of sand, silt, and clay were determined using the Robinson pipette method (Silva 1967; Porta et al. 1986). Soil texture classes were assigned using the USDA soil texture triangle (USDA-NRCS 2018).

References:

LQARS (1977). Methods of Soil Analysis. Laboratório Químico-Agrícola Rebelo da Silva, Lisbon, Portugal.

Porta J, López-Acevedo M, Roquero C (1986) Edafología para la agricultura y el medio ambiente. Ediciones Mundi-Prensa, Madrid

Rodríguez-Echeverría S, Crisóstomo JA, Nabais C, Freitas H (2009) Belowground mutualists and the invasive ability of Acacia longifolia in coastal dunes of Portugal. Biological Invasions 11:651–661. https://doi.org/10.1007/s10530-008-9280-8

Silva AA (1967) Pedologia, Oeiras 2:129–139. In: Silva AA, Alvim AJS, Santos MJ (eds) Métodos de análise de solos, plantas e águas. Estação Agronómica Nacional, Oeiras

USDA-NRCS (2018) Using the soil textural triangle. Soil Science Curriculum. University of Nebraska–Lincoln, Institute of Agriculture and Natural Resources, CropWatch. https://cropwatch.unl.edu/using-soil-textural-triangle

1. **Effect of warming on micro-environmental characteristics**

**Appendix S2.** Model specification of daily mean air temperature

Mean air temperature (MeanT) was analysed using generalized least squares (GLS) models implemented in the **nlme** package in R. To account for the hierarchical structure of the experiment and temporal autocorrelation inherent to daily climatic data, we specified a model including site, treatment (Warming vs Control), and year as predictors, as well as their interactions:

$$\text{MeanT}\boldsymbol{\sim}\text{site}\boldsymbol{\times}\text{treatment}\boldsymbol{\times}\text{year}_{\boldsymbol{c}}\boldsymbol{+}\mathbf{sin}\boldsymbol{(}\boldsymbol{2}\boldsymbol{\pi d}\mathbf{/}\boldsymbol{365}\boldsymbol{)+}\mathbf{cos}\boldsymbol{(}\boldsymbol{2}\boldsymbol{\pi d}\mathbf{/}\boldsymbol{365}\boldsymbol{)}$$

where:

- *site* was included as a fixed factor,
- *treatment* (Warming vs Control) represented the OTC effect,
- *year* was included as a centred continuous covariate (year_c), obtained by subtracting the mean year value from each observation,
- sinusoidal terms (sin1, cos1) modelled seasonal periodicity,
- *d* represents day-of-year.

Mean-centering of year was performed to reduce collinearity among interaction terms and to allow interpretation of main effects at the average year of the study period.

Temporal autocorrelation was modelled using an AR(1) correlation structure within each site × treatment time series:

$$\text{corAR1(form = day | site/treatment)}$$

Additionally, heteroscedasticity among sites was accounted for using a variance structure allowing different residual variances per site:

$$\text{varIdent}\text{(form = 1 | site)}$$

Models were fitted using restricted maximum likelihood (REML). Model diagnostics included inspection of residual distributions, normal Q–Q plots, and autocorrelation functions (ACF) of residuals.

Marginal means and contrasts (warming − control) were estimated using the **emmeans** package to quantify treatment effects overall, by site, and by year. Ninety-five percent confidence intervals were calculated from model-based standard errors.

**Table S2.** Results of generalized least squares (GLS) models testing spatial and temporal variation in the experimental warming effect **on daily mean air temperature**. The response variable represents the daily temperature difference between warming and control plots (ΔT = Warming − Control). Temporal autocorrelation within each site time series was modelled using a first-order autoregressive correlation structure (AR(1)), and heteroscedasticity among sites was accounted for using a site-specific variance structure (varIdent). Year was mean-centred (year_c) to facilitate interpretation of model intercepts. Seasonal variation was modelled using sinusoidal terms (sin1 and cos1). **(A)** Fixed-effect parameter estimates of the GLS model; **(B)** F-tests evaluating the effects of site, year (year_c), seasonal components, and their interaction; **(C)** Site-specific warming effects (ΔT = Warming − Control) estimated using estimated marginal means (emmeans). Values represent the warming effect at the mean year of the study (year_c = 0). Standard errors (SE), 95% confidence intervals, and p-values testing whether ΔT differs from zero are shown.

A) Fixed-effect estimates

| **Predictor** | **Estimate** | **SE** | **t** | **p** |
| --- | --- | --- | --- | --- |
| (Intercept) | 1.92 | 0.062 | 30.80 | <0.001 |
| siteLAC | 0.27 | 0.096 | 2.81 | 0.005 |
| siteCOB | -0.16 | 0.091 | -1.71 | 0.087 |
| siteALX | -0.92 | 0.090 | -10.20 | <0.001 |
| siteCUM | 0.59 | 0.132 | 4.47 | <0.001 |
| year_c | -0.02 | 0.070 | -0.27 | 0.786 |
| sin1 | 0.25 | 0.049 | 4.97 | <0.001 |
| cos1 | -1.05 | 0.045 | -23.56 | <0.001 |
| siteLAC × year_c | 0.04 | 0.105 | 0.39 | 0.695 |
| siteCOB × year_c | -0.19 | 0.099 | -1.96 | 0.050 |
| siteALX × year_c | -0.58 | 0.099 | -5.82 | <0.001 |
| siteCUM × year_c | 0.17 | 0.161 | 1.05 | 0.292 |

B) Tests of fixed effects

| **Term** | **F** | **p** |
| --- | --- | --- |
| site | 62.50 | <0.001 |
| year_c | 2.82 | 0.093 |
| sin1 | 21.26 | <0.001 |
| cos1 | 558.25 | <0.001 |
| site × year_c | 12.64 | <0.001 |

## **C) Site-specific warming effects**

| **Site** | **ΔT (°C)** | **SE** | **95% CI** | **p** |
| --- | --- | --- | --- | --- |
| NSA | 1.92 | 0.062 | 1.79–2.04 | <0.001 |
| LAC | 2.19 | 0.073 | 2.04–2.33 | <0.001 |
| COB | 1.76 | 0.066 | 1.63–1.89 | <0.001 |
| ALX | 1.00 | 0.065 | 0.87–1.13 | <0.001 |
| CUM | 2.51 | 0.117 | 2.28–2.74 | <0.001 |

**
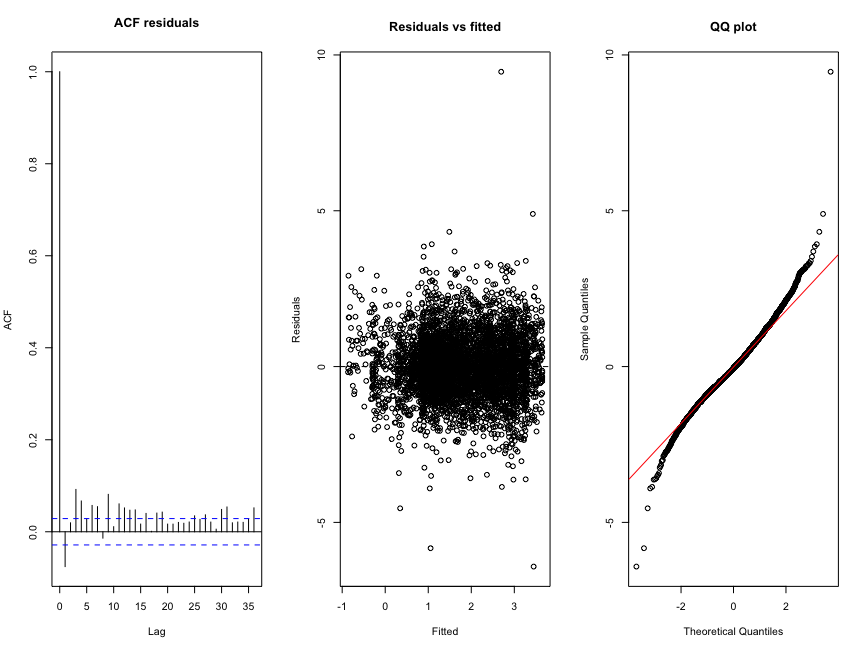
**

**Fig. S1 a) –** Diagnostic plots of the GLS model used to estimate warming effects on daily temperature differences (ΔT). Panels show (A) autocorrelation function (ACF) of normalized residuals, (B) residuals versus fitted values, and (C) normal Q–Q plot. Residual diagnostics indicate no strong remaining temporal autocorrelation and approximately normally distributed residuals.

**
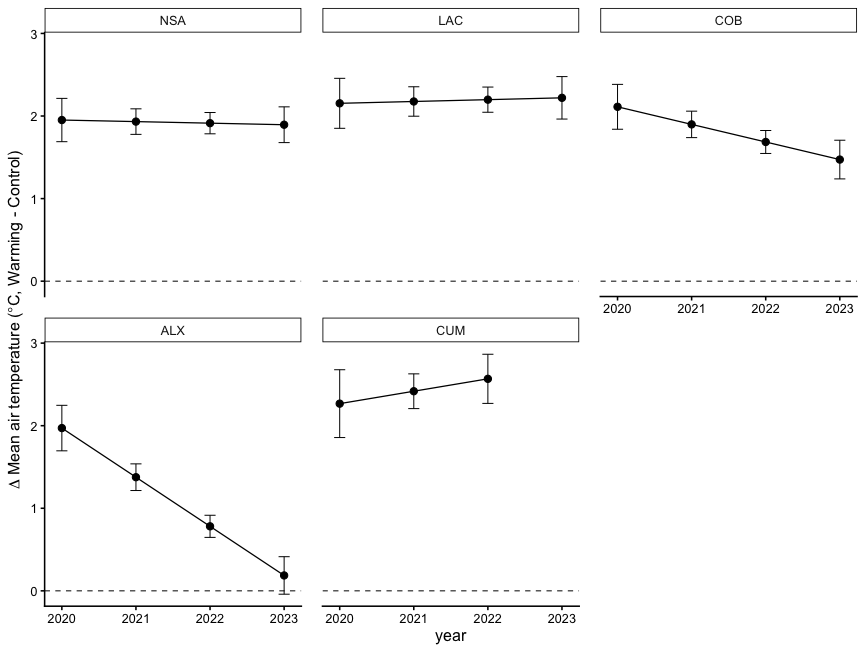
**

**Fig. S1 b) – Site- and year-specific effects of experimental warming (Δ Warming − Control).** Annual estimates of the warming effect on mean air temperature (Δ MeanT, °C; Warming − Control) across study sites. Points represent model estimates derived from a generalized least squares model accounting for temporal autocorrelation (AR1) and seasonal variation (sine and cosine terms). Error bars indicate 95% confidence intervals. The horizontal dashed line denotes no warming effect (Δ = 0). Panels correspond to the five study sites (NSA, LAC, COB, ALX, and CUM). Estimates are shown for the years 2020–2023, except for CUM in 2023 where data were unavailable.


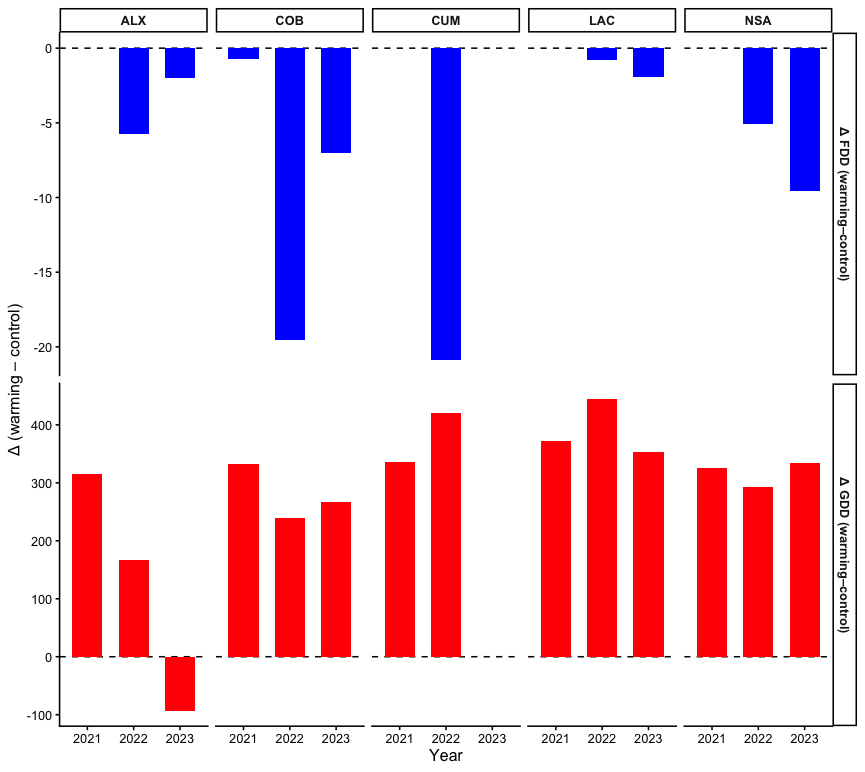


**Fig. S2** **–** Seasonal warming effects on Growing Degree Days (GDD) and Freezing Degree Days (FDD) computed for the meteorological growing season (March–July). Bars show Δ (warming − control) in cumulative degree-days (°C·days) for each site and year. Positive values indicate higher values under warming, whereas negative values indicate lower values compared to control conditions.

**Table S3.** Effects of experimental warming, site, and year on **growing degree days (GDD) and freezing degree days (FDD).** Results of generalized linear models testing the effects of treatment, site, and year on thermal accumulation and freezing exposure accumulated between March and July. GDD was analysed using a Gamma GLM (log link), and FDD using a Tweedie GLMM (log link; glmmTMB). Model explanatory power was high for GDD (Nagelkerke R² = 0.74) and moderate for FDD (McFadden R² = 0.25). The table includes (a) analysis of deviance tables (Type II Wald χ² tests), (b) observed site-specific means and ratios (Warming / Control) with confidence intervals and p-values, and (c) pairwise comparisons of marginal means among years (emmeans, Tukey-adjusted).

1. Analysis of deviance tables

|  |  |  |  | Term | | χ² | | df | | p-value | |
| --- | --- | --- | --- | --- | --- | --- | --- | --- | --- | --- | --- |
| **GDD**  **FDD** | | Treatment | | | 39.15 | | 1 | | <0.0001 | |  |
|  |  | Site | | | 20.00 | | 4 | | 0.0005 | |  |
|  |  | Year | | | 1.33 | | 1 | | 0.249 | |  |
|  |  | Treatment | | | 16.43 | | 1 | | <0.0001 | |  |
|  |  | Site | | | 19.60 | | 4 | | 0.0006 | |  |
|  |  | Year | | | 14.32 | | 1 | | 0.00015 | |  |

# B. Observed mean **GDD** under control and warming treatments at each site.

| Site | Control | Warming | Ratio | **Increase (%)** | 95% CI | p-value |  |
| --- | --- | --- | --- | --- | --- | --- | --- |
| ALX | 783 | 912 | 1.16 | +16.6 | 0.98–1.37 | 0.08 |  |
| COB | 772 | 1052 | 1.37 | +36.2 | 1.15–1.62 | <0.001 |  |
| CUM | 764 | 1142 | 1.50 | +49.4 | 1.22–1.84 | <0.001 |  |
| LAC | 965 | 1355 | 1.40 | +40.4 | 1.19–1.66 | <0.001 |  |
| NSA | 810 | 1128 | 1.40 | +39.3 | 1.18–1.65 | <0.001 |  |

# **C.** Observed mean **FDD** under control and warming treatments at each site.

| Site | Control | Warming | Ratio | **Reduction (%)** | 95% CI |  | p-value |  |
| --- | --- | --- | --- | --- | --- | --- | --- | --- |
| ALX | 3.97 | 1.37 | 0.35 | −65.5 | 0.98–1.37 |  | 0.03 |  |
| COB | 11.1 | 2.00 | 0.18 | −81.9 | 1.15–1.62 |  | <0.001 |  |
| CUM | 12.4 | 2.00 | 0.16 | −83.9 | 1.22–1.84 |  | <0.001 |  |
| LAC | 0.90 | 0.00 | 0.00 | −100 | 1.19–1.66 |  | <0.001 |  |
| NSA | 5.50 | 0.63 | 0.12 | −88.5 | 1.18–1.65 |  | <0.001 |  |

D. Pairwise comparisons of marginal means among years.

|  | Comparison | Difference (degree-days) | SE | | p-value |
| --- | --- | --- | --- | --- | --- |
| **GDD** | 2021–2022 | -108 | 43.4 | | 0.054 |
|  | 2021–2023 | 82 | 42.4 | | 0.155 |
|  | 2022–2023 | 190 | 44.8 | | 0.001 |
| **FDD** | 2021–2022 | -3.21 | | 0.602 | <0.0001 |
|  | 2021–2023 | -3.04 | | 0.651 | <0.0001 |
|  | 2022–2023 | 0.17 | | 0.694 | 0.97 |

**Table S4.** Mean daily ± SE soil volumetric water content % (VWC ± SE) for each **site × year × treatment combination** (n indicates the number of daily sensor observations per group).

| **Site** | **Year** | **Control (n)** | **Control (mean ± SE)** | **Warming (n)** | **Warming (mean ± SE)** |
| --- | --- | --- | --- | --- | --- |
| ALX | 2021 | 115 | 0.619 ± 0.024 | 147 | 0.692 ± 0.013 |
| ALX | 2022 | 222 | 0.615 ± 0.017 | 277 | 0.618 ± 0.015 |
| ALX | 2023 | 250 | 0.580 ± 0.013 | 216 | 0.617 ± 0.017 |
| COB | 2021 | 163 | 0.375 ± 0.015 | 150 | 0.446 ± 0.019 |
| COB | 2022 | 345 | 0.396 ± 0.012 | 293 | 0.500 ± 0.015 |
| COB | 2023 | 245 | 0.329 ± 0.011 | 268 | 0.457 ± 0.012 |
| CUM | 2021 | 142 | 0.435 ± 0.022 | 126 | 0.455 ± 0.024 |
| CUM | 2022 | 288 | 0.464 ± 0.015 | 182 | 0.511 ± 0.018 |
| LAC | 2021 | 168 | 0.382 ± 0.011 | 167 | 0.294 ± 0.012 |
| LAC | 2022 | 365 | 0.442 ± 0.008 | 364 | 0.327 ± 0.008 |
| LAC | 2023 | 282 | 0.453 ± 0.009 | 272 | 0.253 ± 0.007 |
| NSA | 2021 | 167 | 0.478 ± 0.019 | 168 | 0.304 ± 0.011 |
| NSA | 2022 | 362 | 0.573 ± 0.012 | 365 | 0.300 ± 0.006 |
| NSA | 2023 | 281 | 0.486 ± 0.010 | 282 | 0.331 ± 0.007 |

**Table S5.** Results of the generalized least squares (GLS) model testing the effects of site, treatment, and their interaction on **daily soil moisture** (volumetric water content, VWC%), with year included as a mean-centred continuous covariate (year_c). Soil moisture was analysed using 6,672 daily observations across all site × treatment combinations. Temporal autocorrelation in daily sensor measurements was accounted for using a first-order autoregressive correlation structure (AR1), and heteroscedasticity among sites was accommodated using a site-specific variance structure (varIdent). The table includes: (A) Type III ANOVA results for fixed effects in the GLS model. (B) Estimated warming effects on soil moisture for each site (ΔVWC = Warming − Control). Values are expressed as percentage differences derived from estimated marginal means of the GLS model.

a)

| Effect | df | F-value | p-value |
| --- | --- | --- | --- |
| (Intercept) | 1 | 735.2931 | <0.0001 |
| site | 4 | 4.5946 | 0.0011 |
| treatment | 1 | 14.7202 | 0.0001 |
| year_c | 1 | 0.8064 | 0.3692 |
| site:treatment | 4 | 3.5357 | 0.0069 |

b)

| **Site** | **Δ VWC (%)** | **95% CI (lower)** | **95% CI (upper)** | **p-value** |
| --- | --- | --- | --- | --- |
| ALX | +4.1 | −14.9 | 23.0 | 0.67 |
| COB | +10.1 | −8.1 | 28.3 | 0.27 |
| CUM | +1.0 | −26.9 | 29.0 | 0.94 |
| LAC | −13.9 | −32.3 | 4.5 | 0.14 |
| NSA | −20.6 | −28.9 | −12.3 | <0.001 |

1. **Effect of warming on plant diversity, growth and nutrient content**

**Table S6**. Mean (± SE) **plant diversity indices** (species richness, Shannon diversity, inverse Simpson diversity and Pielou evenness) for each site × treatment combination. Values are calculated from plot-level data.

| **Site** | **treatment** | **Richness** | **Shannon** | **Inverse Simpson** | **Pielou** | **n** |
| --- | --- | --- | --- | --- | --- | --- |
| ALX | Control | 4.47 ± 0.32 | 0.668 ± 0.085 | 1.60 ± 0.12 | 0.452 ± 0.049 | 15 |
| ALX | Warming | 6.47 ± 0.36 | 0.880 ± 0.073 | 1.79 ± 0.13 | 0.477 ± 0.039 | 15 |
| COB | Control | 4.20 ± 0.28 | 0.947 ± 0.059 | 2.23 ± 0.14 | 0.676 ± 0.033 | 15 |
| COB | Warming | 5.00 ± 0.37 | 0.872 ± 0.068 | 1.92 ± 0.13 | 0.555 ± 0.037 | 15 |
| CUM | Control | 6.00 ± 0.45 | 0.952 ± 0.057 | 2.00 ± 0.13 | 0.551 ± 0.042 | 10 |
| CUM | Warming | 7.00 ± 0.37 | 0.882 ± 0.040 | 1.66 ± 0.05 | 0.458 ± 0.019 | 10 |
| LAC | Control | 6.60 ± 0.77 | 0.843 ± 0.099 | 1.75 ± 0.13 | 0.486 ± 0.047 | 15 |
| LAC | Warming | 7.47 ± 0.53 | 0.930 ± 0.088 | 1.83 ± 0.15 | 0.467 ± 0.038 | 15 |
| NSA | Control | 3.27 ± 0.42 | 0.468 ± 0.101 | 1.42 ± 0.13 | 0.443 ± 0.051 | 15 |
| NSA | Warming | 4.00 ± 0.37 | 0.582 ± 0.076 | 1.45 ± 0.09 | 0.418 ± 0.036 | 15 |

**Table S7**. Results of linear mixed-effects models (LMMs) for plant diversity indices. Models included site, year, and treatment as fixed effects and plot as a random intercept. The site × treatment interaction was tested but excluded from the final models because it was not significant and did not improve model fit. Specifically, the interaction between warming treatment and site was not significant for species richness (F₄,40.5 = 0.28, p = 0.891) or inverse Simpson diversity (F₄,42.5 = 1.54, p = 0.209), indicating that warming effects on diversity were consistent across sites. The table includes: (A) Type III ANOVA results (df, F-values, and p-values) for all diversity indices; marginal and conditional R² and variance components of the random intercept (plot) are also reported for each model. (B) Estimated marginal means and treatment contrasts for species richness. (C) Tukey-adjusted pairwise comparisons among sites. (D) Pairwise comparisons among years estimated using *emmeans*.

A.

| **Index** | **Factor** | **Df (NumDF)** | | **F value** | | | **Pr(>F)** |  |  |
| --- | --- | --- | --- | --- | --- | --- | --- | --- | --- |
| Plant species richness | Treatment | 44.50 | | 6.23 | | **0.016** | | | |
|  | Site | 44.80 | | 8.31 | | <0.001 | | | |
|  | Year | 88.56 | | 1.27 | | 0.285 | | | |
|  | R^2^m: 0.40; R^2^c: 0.80 |  | |  | |  | | | |
|  | SD plot intercept = 1.41, residual SD = 097. | | | | | | | | |
| Shannon Diversity | Treatment | 42.16 | 0.804 | | 0.375 | | | | |
|  | Site | 42.25 | 7.213 | | <0.0001 | | | | |
|  | Year | 89.82 | 36.18 | | <0.0001 | | | | |
|  | R^2^m: 0.39; R^2^c: 0.65 | | | | | | | |  |
|  | SD plot intercept = 0.17, residual SD = 0.21. | | | | | | | |  |
| Inverse Simpson (log) | Treatment | 44.94 | 0.147 | | 0.754 | | | | |
|  | Site | 45.93 | 7.781 | | <0.0001 | | | | |
|  | Year | 89.46 | 32.13 | | <0.0001 | | | | |
|  | R^2^m: 0.39; R^2^c: 0.56 | | | | | | | |  |
|  | SD plot intercept = 0.05, residual SD = 0.08. | | | | | | | |  |

| Pielou Evenness | Treatment | 43.28 | 3.699 | **0.061** |
| --- | --- | --- | --- | --- |
|  | Site | 44.79 | 8.559 | <0.0001 |
|  | Year | 87.49 | 40.21 | <0.0001 |
|  | R^2^m: 0.28; R^2^c: 0.52 | | | |
|  | SD plot intercept = 0.034, residual SD = 0.12. | | | |

|  |  |  |  |  |  |
| --- | --- | --- | --- | --- | --- |

B. Plant species richness

| **Treatment** | **Mean ± SE** | **95% CI** |  |
| --- | --- | --- | --- |
| Control (C) | 4.92 ± 0.31 | 4.30–5.54 |  |
| Warming (W) | 6.00 ± 0.31 | 5.38–6.62 |  |
| Contrast: W-C = **+1.08**, p = 0.016 | | | |

C. Pairwise comparisons among sites**.** Different letters indicate significant differences among sites based on Tukey-adjusted pairwise comparisons (p < 0.05) within each index.

| **Site** | **Richness (±SE)** | **Shannon (±SE)** | **Inverse Simpson (±SE)** | **Pielou (±SE)** |
| --- | --- | --- | --- | --- |
| ALX | 5.47 ± 0.48 **abc** | 0.774 ± 0.065 **ab** | 0.213 ± 0.022 **bc** | 0.465 ± 0.025 **bc** |
| COB | 4.60 ± 0.48 **bc** | 0.909 ± 0.065 **a** | 0.303 ± 0.022 **a** | 0.616 ± 0.025 **a** |
| CUM | 6.57 ± 0.50 **ab** | 0.988 ± 0.072 **a** | 0.282 ± 0.025 **ab** | 0.549 ± 0.030 **ab** |
| LAC | 7.03 ± 0.48 **a** | 0.887 ± 0.065 **a** | 0.235 ± 0.022 **ab** | 0.476 ± 0.025 **bc** |
| NSA | 3.63 ± 0.48 **c** | 0.525 ± 0.065 **b** | 0.142 ± 0.022 **c** | 0.431 ± 0.026 **c** |

| D. Pairwise comparisons across years*.* | | | | | | | | | |
| --- | --- | --- | --- | --- | --- | --- | --- | --- | --- |
| **Index** | **Contrast - Year** | **Estimate** | | **SE** | | **DF** | | **P-value** | |
| Inverse simpson | 2021 - 2022  **2021 - 2023**  2022 - 2023 | 0.1021 | 0.0166 | | 88.3 | | <0.0001 | |  |
|  |  | -0.0315 | 0.0181 | | 88.3 | | 0.1952 | |  |
|  |  | -0.1336 | 0.0181 | | 88.3 | | <0.0001 | |  |

| Shannon | 2021 - 2022  **2021 - 2023**  2022 - 2023 | 0.271 | 0.0411 | 88.2 | <0.0001 |
| --- | --- | --- | --- | --- | --- |
|  |  | -0.079 | 0.0448 | 88.2 | 0.1918 |
|  |  | -0.349 | 0.0448 | 88.2 | <0.0001 |
| Evenness | 2021 - 2022  **2021 - 2023**  2022 - 2023 | 0.164 | 0.0239 | 86.6 | <0.0001 |
|  |  | -0.052 | 0.0261 | 86.6 | 0.1175 |
|  |  | -0.216 | 0.0261 | 86.6 | <0.0001 |

**Table S8.** Mean aboveground plant biomass (g m⁻²) per site, year and treatment. Values represent mean ± SE (n = 5 plots per treatment).

| **Site** | **Year** | **Control (mean ± SE)** | **Warming (mean ± SE)** |
| --- | --- | --- | --- |
|  | 2021 | 195.70 ± 16.20 | 321.96 ± 11.58 |
| **ALX** | 2022 | 125.65 ± 7.51 | 186.14 ± 11.15 |
|  | 2023 | 111.32 ± 10.02 | 166.46 ± 22.58 |
|  | 2021 | 188.62 ± 30.33 | 276.73 ± 34.51 |
| **COB** | 2022 | 58.44 ± 12.82 | 143.76 ± 23.52 |
|  | 2023 | 85.20 ± 12.75 | 110.79 ± 21.31 |
| **CUM** | 2021 | 151.97 ± 17.43 | 153.85 ± 13.66 |
|  | 2022 | 61.61 ± 16.65 | 89.94 ± 8.47 |
|  | 2021 | 164.93 ± 16.92 | 151.38 ± 19.58 |
| **LAC** | 2022 | 109.53 ± 16.18 | 87.09 ± 10.26 |
|  | 2023 | 114.78 ± 7.34 | 106.34 ± 14.26 |
|  | 2021 | 217.41 ± 32.85 | 300.35 ± 31.06 |
| **NSA** | 2022 | 148.68 ± 19.75 | 183.43 ± 9.41 |
|  | 2023 | 77.53 ± 11.35 | 82.36 ± 23.39 |

**Table S9.** Linear mixed-effects model testing the effects of experimental warming on log₁₀-transformed **plant biomass**. The model included treatment, site, year, and the treatment × site interaction as fixed effects, with plot included as a random intercept. An interaction between treatment and year was initially tested but was not significant (p = 0.18) and did not improve model fit; therefore, it was excluded from the final model. (A) Type III ANOVA table (Satterthwaite approximation) for fixed effects. (B) Estimated treatment effects within each site (emmeans; Kenward–Roger degrees of freedom). (C) Pairwise comparisons between years (emmeans contrasts; Tukey-adjusted p-values).

A.

| **Effect** | **NumDF** | **DenDF** | **F** | **p** |
| --- | --- | --- | --- | --- |
| Treatment | 1 | 41.47 | 12.06 | 0.001 |
| Site | 4 | 40.87 | 8.95 | <0.001 |
| Year | 2 | 88.46 | 66.70 | <0.001 |
| Treatment × Site | 4 | 40.23 | 2.67 | 0.046 |

| **Random factor** | **Variance** | **SD** |
| --- | --- | --- |
| site:plot (intercept) | 0.0043 | 0.07 |
| Residual | 0.0220 | 0.15 |

| R² marginal (fixed effects) | 0.56 |
| --- | --- |
| R² conditional (fixed + random) | 0.63 |
| n (observations) | 140 |
| n (plots) | 50 |

B.

| **Site** | **Estimate** | **SE** | **df** | **p** | **% change** |
| --- | --- | --- | --- | --- | --- |
| ALX | 0.186 | 0.068 | 36.6 | 0.010 | +53% |
| COB | 0.227 | 0.068 | 36.6 | 0.002 | +69% |
| CUM | 0.115 | 0.078 | 61.8 | 0.297 | +30% |
| LAC | -0.060 | 0.068 | 36.6 | 0.381 | −13% |
| NSA | 0.079 | 0.068 | 36.6 | 0.257 | +20% |

C.

| **Contrast** | **Estimate (log10)** | **SE** | **df** | **t** | **p-value** | **% change** |
| --- | --- | --- | --- | --- | --- | --- |
| 2021−2022 | 0.2725 | 0.0297 | 86.4 | 9.183 | <0.0001 | +87.3% |
| 2021−2023 | 0.3421 | 0.0323 | 86.4 | 10.579 | <0.0001 | +119.8% |
| 2022−2023 | 0.0696 | 0.0323 | 86.4 | 2.152 | 0.0854 | +17.4% |

**Figure S3.** Standardized effect sizes (Hedges’ g ± 95% CI) describing the magnitude of warming effects on plant species richness and aboveground biomass for each study year. Effect sizes were calculated using site-level means as replicates (n = 5 sites per year; n = 4 in 2023 after the loss of CUM). Positive values indicate higher values under warming relative to control.

**Table S10.** Summary of linear (LM) and generalized linear (GLM) models assessing the effects of the warming treatment (OTC), site, and their interaction on Nardus stricta leaf nutrient concentrations (%) measured at the end of the 2022 growing season. The carbon (C) model was fitted using a Gamma GLM with a log link. Marginal R² values represent the proportion of variance explained by fixed effects. Estimated marginal means for the C model are presented on the response scale. (A) Type II/III ANOVA results for nutrient models (N, C, C:N, and P). (B1) Estimated marginal means of leaf N and C:N by treatment. (B2) Estimated marginal means of leaf N, C, C:N, and P by site (averaged across treatments).

A.

| **Response** | Term | Df | Test statistic | p |
| --- | --- | --- | --- | --- |
| **N (%)** | Treatment | 1 | F = 19.17 | <0.0001 |
|  | Site | 4 | F = 7.93 | <0.0001 |
|  | Treatment × Site | 4 | F = 2.19 | 0.087 |
|  | Residuals | 40 |  |  |
|  | **R²** |  | **0.55** |  |
| **C:N** | Treatment | 1 | F = 16.86 | 0.0002 |
|  | Site | 4 | F = 13.72 | <0.0001 |
|  | Treatment × Site | 4 | F = 1.80 | 0.147 |
|  | Residuals | 40 |  |  |
|  | **R²** |  | **0.62** |  |
| **P (mg kg⁻¹)** | Treatment | 1 | F = 0.84 | 0.365 |
|  | Site | 4 | F = 8.09 | <0.0001 |
|  | Treatment × Site | 4 | F = 0.57 | 0.686 |
|  | Residuals | 40 |  |  |
|  | **R²** |  | **0.42** |  |
| **C (%)** | Treatment | 1 | χ² = 1.53 | 0.215 |
|  | Site | 4 | χ² = 4.18 | 0.382 |
|  | Treatment × Site | 4 | χ² = 4.78 | 0.311 |

B1) Estimated marginal means of leaf N (%) and C:N ratio by treatment

|  | Control | Warming | Estimate (Warming -Control) | *p* |
| --- | --- | --- | --- | --- |
| **N** | 1.51± 0.017 | 1.40 ± 0.017 | -0.103 | <0.0001 |
| **C:N** | 29.00 ± 0.32 | 30.84 ± 0.32 | 1.835 | 0.0002 |

##

## **B2)** Estimated marginal means (± SE) of leaf nutrient traits by site (averaged across treatments)

| **Site** | **N (%)** | **C (%)** | **C:N** | **P (mg kg⁻¹)** |
| --- | --- | --- | --- | --- |
| ALX | 1.53 ± 0.03 **b** | 42.85 ± 0.50 | 28.09 ± 0.50 **a** | 1628 ± 73 **bc** |
| COB | 1.52 ± 0.03 **b** | 43.41 ± 0.50 | 28.70 ± 0.50 **a** | 1725 ± 73 **c** |
| CUM | 1.44 ± 0.03 **ab** | 42.93 ± 0.50 | 29.83 ± 0.50 **a** | 1218 ± 73 **a** |
| LAC | 1.43 ± 0.03 **ab** | 42.87 ± 0.50 | 30.10 ± 0.50 **a** | 1576 ± 73 **bc** |
| NSA | 1.35 ± 0.03 **a** | 44.03 ± 0.51 | 32.89 ± 0.50 **b** | 1364 ± 73 **ab** |

### **Note:** Site means are averaged across treatments because the Treatment × Site interaction was not significant. Carbon (C) showed no significant site effect; therefore, no post-hoc comparisons were performed. Different letters indicate significant differences among sites within each nutrient variable based on Sidak-adjusted pairwise comparisons (p < 0.05).

**Table S11.** **Beta diversity** based on Jaccard dissimilarity between control and warming plots within each year (A) and within each site and year (B). Total dissimilarity (β.jac) is split into turnover (β.jtu: species replacement) and nestedness (β.jne: species loss/gain without replacement). Higher values indicate greater compositional differences between treatments.

A.

| **Year** | **Total** | **Turnover** | **Nestedness** |
| --- | --- | --- | --- |
| 2021 | 0.589 | 0.272 | 0.317 |
| 2022 | 0.591 | 0.31 | 0.281 |
| 2023 | 0.579 | 0.257 | 0.322 |

B.

| **Site** | **Year** | **Total** | **Turnover** | **Nestedness** |
| --- | --- | --- | --- | --- |
| **ALX** | 2021 | 0.653 | 0.359 | 0.294 |
|  | 2022 | 0.596 | 0.29 | 0.306 |
|  | 2023 | 0.599 | 0.261 | 0.338 |
| **COB** | 2021 | 0.517 | 0.244 | 0.273 |
|  | 2022 | 0.530 | 0.248 | 0.282 |
|  | 2023 | 0.435 | 0.155 | 0.280 |
| **CUM** | 2021 | 0.450 | 0.182 | 0.268 |
|  | 2022 | 0.608 | 0.378 | 0.230 |
| **LAC** | 2021 | 0.702 | 0.387 | 0.314 |
|  | 2022 | 0.750 | 0.469 | 0.280 |
|  | 2023 | 0.638 | 0.354 | 0.284 |
| **NSA** | 2021 | 0.623 | 0.188 | 0.436 |
|  | 2022 | 0.472 | 0.166 | 0.306 |
|  | 2023 | 0.643 | 0.258 | 0.385 |

**Table S12.** Site-specific responses (Δ cover) and standardized effect sizes (Hedges’ g) for dominant plant species *Nardus stricta* and other non-dominant species with sufficient replication to estimate variance. Hedges’ g represents the bias-corrected standardized mean difference between Warming and Control treatments, calculated separately for each site × year using plot-level replication (n = 5 plots per treatment). For each species, gₘₑₐₙ represents the mean effect size across years. Effect sizes were calculated only when species (i) occurred in both treatments within a site × year combination, (ii) showed non-zero variance in at least one treatment group, and (iii) allowed estimation of the pooled standard deviation. Species present in fewer years are included when effect sizes could be estimated but should be interpreted cautiously due to lower temporal replication.

| Site | *Species* | Year (n) | **Δ cover (mean)** | **ΔSE** | **Hedges’ g (mean)** | status |
| --- | --- | --- | --- | --- | --- | --- |
|  | ***Nardus stricta*** | **3** | **1.27** | **3.820** | **0.203** | **Increased** |
| **ALX** | *Aulacomnium palustre* | 2 | −0.60 | 0.40 | -1.06 | Lost |
|  | *Festuca rothmaleri* | 3 | 1.13 | 0.98 | 0.16 | Increased |
|  | *Carex nigra* | 3 | 0.53 | 0.07 | 0.28 | Gained |
|  | *Potentilla erecta* | 3 | 1.67 | 1.96 | 0.29 | Increased |
|  | *Juncus squarrosus* | 3 | 0.406 | 0.222 | 0.662 | Increased |
|  | *Sphagnum sp.* | 1 | −0.80 | NA | NA | Lost |
|  | ***Nardus stricta*** | **3** | **10.2** | **4.47** | **0.393** | **Increased** |
| **COB** | *Festuca henriquesii* | 3 | −6.47 | 1.67 | −0.20 | Decreased |
|  | *Festuca rothmaleri* | 2 | 1.30 | 1.10 | 0.15 | Increased |
|  | *Juncus squarrosus* | 3 | 2.00 | 0.58 | 0.45 | Increased |
|  | *Potentilla erecta* | 3 | −2.87 | 1.68 | −0.37 | Decreased |
|  | *Gallium saxatile* | 3 | 0.20 | 0.63 | 0.14 | Increased |
|  | ***Nardus stricta*** | **2** | **22.4** | **2.99** | **1.26** | **Increased** |
| **CUM** | *Agrostis truncatula* | 2 | 2.60 | 0.80 | 0.71 | Increased |
|  | *Gentiana pneumonanthe* | 2 | 0.60 | 0.30 | 0.23 | Increased |
|  | *Juncus squarrosus* | 2 | −15.30 | 2.50 | −1.33 | Decreased |
|  | *Hieracium sp.* | 2 | -0.30 | 0.3 | -0.40 | Decreased |
|  | *Narcissus bulbocodium* | 1 | −1.80 | 1.93 | -0.53 | Decreased |
|  | *Molineriella laevis* | 1 | 1.4 | NA | NA | Increased |
|  | ***Nardus stricta*** | **3** | **2.47** | **1.25** | **0.254** | **Increased** |
| **LAC** | *Arenaria montana* | 3 | −1.73 | 0.71 | −0.55 | Decreased |
|  | *Armeria sampaioi* | 3 | 0.60 | 0.31 | 0.90 | Gained |
|  | *Hypochaeris radicata* | 3 | 1.00 | 0.81 | 0.40 | Increased |
|  | *Lotus corniculatus* | 2 | 4.80 | 1.62 | 1.16 | Increased |
|  | *Ranunculus bulbosus* | 2 | −0.60 | 0.35 | −0.70 | Decreased |
|  | *Orthotrichum stramineum* | 1 | 0.6 | NA | NA | Gained |
|  | ***Nardus stricta*** | **3** | **2.75** | **1.75** | **0.18** | **Increased** |
| **NSA** | *Calluna vulgaris* | 2 | 2.43 | 0.79 | 0.51 | Increased |
|  | *Erica arborea* | 1 | 2.30 | NA | NA | Gained (2023) |
|  | *Festuca rothmaleri* | 2 | 0.43 | 0.83 | 0.02 | Increased |
|  | *Narcissus sp.* | 3 | −1.19 | 0.13 | -0.6 | Decreased |
|  | *Pedicularis sylvatica* | 2 | -0.68 | 0.08 | -0.33 | Decreased |

**Appendix S3. Formulas and indices used**

**1. Jaccard-based Dissimilarity Partitioning (Baselga 2010, 2012)**

Given two assemblages (Warming vs. Control):

**a** = number of species shared between assemblages

**b** = number of species unique to the first assemblage

**c** = number of species unique to the second assemblage

**Total Jaccard dissimilarity (β jac) -** overall dissimilarity between two assemblages.

**
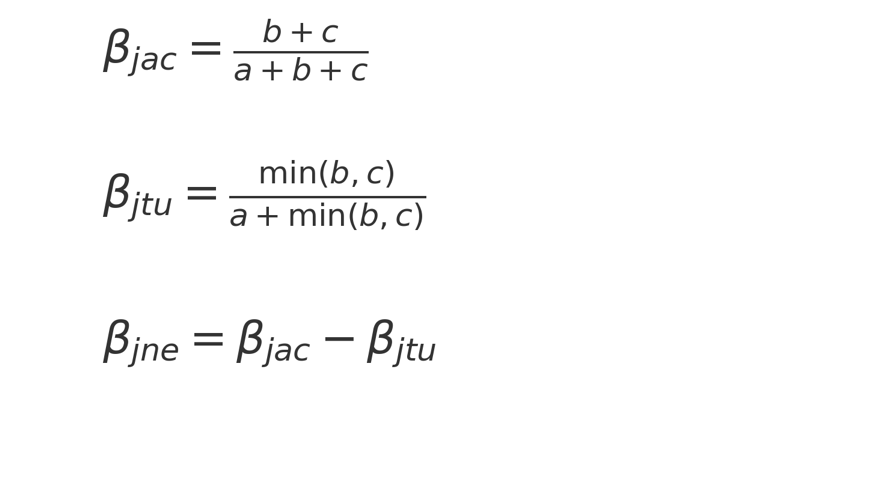
**

**Turnover component (βjtu) -** species replacement between assemblages.

**
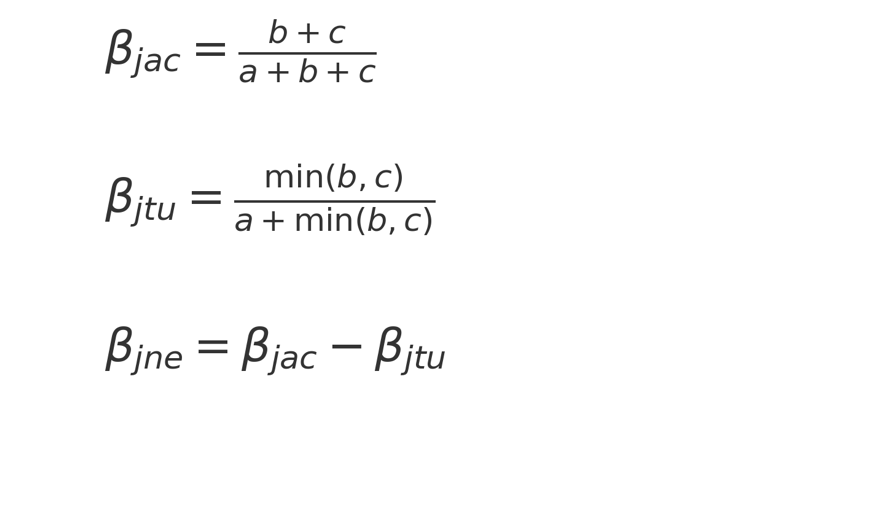
**

**Nestedness-resultant component (βjne) -** differences due to species loss or gain.

**
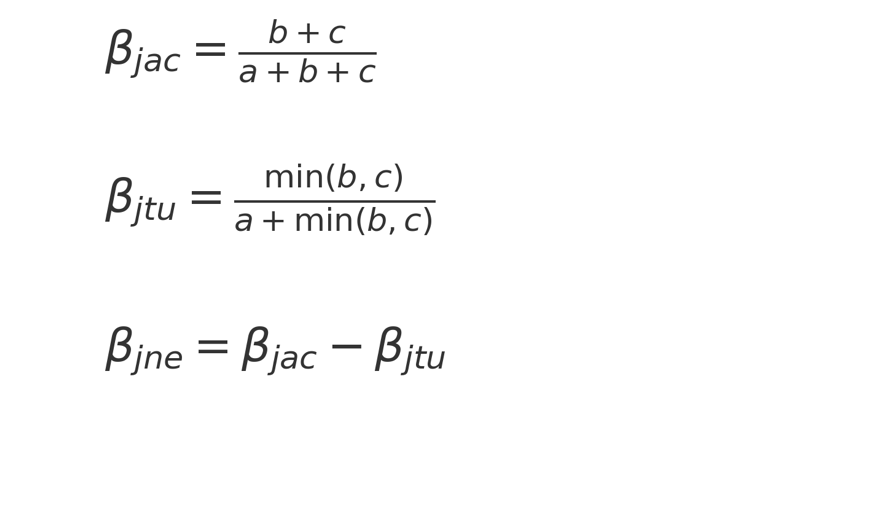
**

## **2. Standardized effect size calculation (Hedges’ g)**

To summarize the magnitude of ecological responses to experimental warming, we calculated standardized effect sizes (Hedges’ g), a bias-corrected standardized mean difference expressing treatment effects relative to pooled variability (Hedges & Olkin 1985; Nakagawa & Cuthill 2007). Effect sizes were calculated for aboveground biomass, plant species richness, and individual plant species responses.

**g =** J × (X̄W − X̄C) / Sp

where X̄W and X̄C are the mean values for the warming and control treatments, respectively, and Sp is the pooled standard deviation:

**Sp =** √[ ((nW − 1)SW² + (nC − 1)SC²) / (nW + nC − 2) ]

where SW and SC represent the standard deviations for the warming and control treatments, and nW and nC are the corresponding sample sizes.

To correct for small sample bias, the standardized mean difference was multiplied by the correction factor J:

**J =** 1 − 3 / (4(nW + nC) − 9)

The sampling variance of Hedges’ g was estimated as:

**Var(g) =** (nW + nC)/(nW nC) + g² / [2(nW + nC − 2)]

This variance estimate was used to calculate confidence intervals and, when appropriate, to derive inverse‑variance weighted effect size estimates.

Positive values of g indicate higher values under warming relative to control conditions.

Effect sizes were interpreted following commonly used thresholds: very small (|g| < 0.2), small (0.2 ≤ |g| < 0.5), medium (0.5 ≤ |g| < 0.8), large (0.8 ≤ |g| < 1.2), and very large (|g| ≥ 1.2).
